# Supplementary material for: Rhodium nanoparticles supported on silanol-rich zeolites beyond the homogeneous Wilkinson’s catalyst for hydroformylation of olefins
Source: Nat Commun. 2023 May 3;14:2531. doi: 10.1038/s41467-023-38181-6 (PMC10156763; doi:10.1038/s41467-023-38181-6)
Supplement: Supplementary file 1 — Supplementary Information [file 41467_2023_38181_MOESM1_ESM.pdf]

Supplementary Information

Rhodium nanoparticles supported on silanol-rich zeolites beyond the homogeneous Wilkinson's catalyst for hydroformylation of olefins

Yifeng Liu,[1#] Zhiqiang Liu,[2,#] Yu Hui,[3#] Liang Wang,[1,*] Jian Zhang,[4] Xianfeng Yi,[2] Wei Chen[2], Chengtao Wang,[1] Hai Wang,[1] Yucai Qin,[3] Lijuan Song,[3] Anmin Zheng,[2] and Feng-Shou Xiao[1,4,*]

[1] Key Lab of Applied Chemistry of Zhejiang Province and Department of Chemistry & Key Lab of Biomass Chemical Engineering of Ministry of Education and College of Chemical and Biological Engineering, Zhejiang University, Hangzhou 310027, China.

[2] National Center for Magnetic Resonance in Wuhan, State Key Laboratory of Magnetic Resonance and Atomic and Molecular Physics and Mathematics, Wuhan Institute of Physics and Mathematics, Innovation Academy for Precision Measurement Science and Technology, Chinese Academy of Sciences, Wuhan 430071, China.

[3] Key Laboratory of Petrochemical Catalytic Science and Technology, Liaoning Shihua University, Fushun 113001, China.

[4] Beijing Advanced Innovation Center for Soft Matter, Science and Engineering, Beijing University of Chemical Technology, Beijing 100029, China.

*Correspondence to: liangwang@zju.edu.cn; fsxiao@zju.edu.cn

[#]These authors contributed equally.

Table of Contents

Supplementary Methods

Supplementary Fig. 1. XRD patterns of the (a) S1-OH and (b) Rh/S1-OH.

Supplementary Fig. 2. N_2 sorption isotherms of the (a) S1-OH and (b) Rh/S1-OH samples.

Supplementary Fig. 3. TEM images of the (a) S1-OH and (b) Rh/S1-OH samples.

Supplementary Fig. 4. SEM images of the (a) S1-OH and (b) Rh/S1-OH samples.

Supplementary Fig. 5. (a) XRD pattern, (b) N_2 sorption isotherms, and (c) SEM image of the S-1 sample.

Supplementary Fig. 6. ^{29}Si MAS NMR spectra of S1-OH and S-1 zeolites.

Supplementary Fig. 7. Scheme showing the structure of different silanols in zeolites.

Supplementary Fig. 8. (a) XRD pattern, (B) N_2 sorption isotherms, and (c) SEM image of the Rh/S-1 sample.

Supplementary Fig. 9. (a) TEM image and (b) Rh nanoparticle size distribution of the Rh/S-1 sample.

Supplementary Fig. 10. (a) Tomographic section TEM image and (b) Rh nanoparticle size distribution of the Rh/S1-OH samples.

Supplementary Fig. 11. Rh dispersions of the (a) Rh/S1-OH and (b) Rh/S-1 samples.

Supplementary Fig. 12. Rh *3d* XPS spectra of the Rh/S1-OH and Rh/S-1 samples.

Supplementary Fig. 13. GC profiles analyzing the products from (a) S1-OH and (b) Rh/S1-OH catalyzed styrene hydroformylation.

Supplementary Fig. 14. (a) XRD pattern, (b) N_2 sorption isotherms, and (c) TEM image of the Rh/ZSM-5 catalyst.

Supplementary Fig. 15. (a) XRD pattern and (b) TEM image of the Rh/SiO_2 catalyst.

Supplementary Fig. 16. (a) XRD pattern and (b) TEM image of the Rh/γ-Al_2O_3 catalyst.

Supplementary Fig. 17. (a) XRD pattern and (b) TEM image of the Rh/TiO_2 catalyst.

Supplementary Fig. 18. (a) Structural formula, (b) ^{1}H, (c) ^{13}C, and (d) ^{31}P NMR spectra of Wilkinson's catalyst.

Supplementary Fig. 19. (a) Data characterizing the performances of the Rh/S1-OH in the recycle tests for styrene hydroformylation. (b) Reaction rate of the Rh/S1-OH in the recycle tests for styrene hydroformylation.

Supplementary Fig. 20. (a) TEM images and (b) Rh nanoparticle size distribution of the used Rh/S1-OH catalyst after the recycle tests.

Supplementary Fig. 21. Data showing the styrene conversion in the standard reaction and hot-filtration reaction.

Supplementary Fig. 22. XRD patterns of the Rh/S1-OH-5, Rh/S1-OH-10, and Rh/S1-OH-20 samples.

Supplementary Fig. 23. N_2 sorption isotherms of the (a) Rh/S1-OH-5, (b) Rh/S1-OH-10, and (c) Rh/S1-OH-20 samples.

Supplementary Fig. 24. SEM images of the (a) Rh/S1-OH-5, (b) Rh/S1-OH-10, and (c) Rh/S1-OH-20 samples.

Supplementary Fig. 25. TEM images of the (a) Rh/S1-OH-5, (b) Rh/S1-OH-10, and (c) Rh/S1-OH-20.

Supplementary Fig. 26. Rh *3d* XPS spectra of the Rh/S1-OH-5, Rh/S1-OH-10, and Rh/S1-OH-20 samples.

Supplementary Fig. 27. (a) 2D ^{1}H–^{1}H DQ MAS NMR of the Rh/S1-OH-5 sample. (b)^{1}H MAS NMR of the Rh/S1-OH-5, Rh/S1-OH-10, and Rh/S1-OH-20 samples.

Supplementary Fig. 28. (a) ^{29}Si MAS NMR spectra of Rh/S1-OH-5, Rh/S1-OH-10, and Rh/S1-OH-20 samples. Inset in figure, ^{29}Si CP/MAS NMR spectra of the three samples. (b) FTIR spectra of the Rh/S1-OH-5, Rh/S1-OH-10, and Rh/S1-OH-20 samples.

Supplementary Fig. 29. (a) XRD patterns and SEM images of the (b) Rh/MFI-deGa-30 and (c) Rh/MFI-deGa-60 catalysts.

Supplementary Fig. 30. Data characterizing the catalytic performances of the Rh/MFI-deGa-30 and Rh/MFI-deGa-60 catalysts in the hydroformylation of styrene.

Supplementary Fig. 31. Step-dosing experiments over the (a) Rh/S1-OH, (b) Rh/S-1, and (c) Rh/SiO_2 catalysts in ethylene hydrogenation.

Supplementary Fig. 32. Arrhenius plots of styrene hydroformylation over the Rh/S1-OH and Rh/S-1 catalysts.

Supplementary Fig. 33. Kinetic reaction orders to (a) hexene and (b) ethylene in Rh/S-1 and Rh/S1-OH catalyzed hydroformylation.

Supplementary Fig. 34. Temperature-programmed desorption tests of ethylene on the Rh/S1-OH and Rh/S-1 samples.

Supplementary Fig. 35. *In situ* hexene-adsorption FTIR spectra over the Rh/S1-OH catalyst.

Supplementary Fig. 36. Initial structure of 100 ethylene molecules (C atoms in orange, H atoms in white) adsorbed in gas phase near the S1-OH zeolite surface (grey framework with hydroxyl group in red-white).

Supplementary Fig. 37. Percentage of ethylene molecules in free region (simulating the homogeneous system) and S1-OH zeolite during the diffusion process with the initial number of ethylene molecules at (a) 50 and (b) 200.

Supplementary Fig. 38. Periodic structure of ethylene in (a) free region (simulating the homogeneous system, 120.5 × 120.0 × 105.1 $Å^3$) and (b) S1-OH zeolite (60.3 × 59.2 × 52.6 $Å^3$).

Supplementary Fig. 39. Distribution of two nearest-neighbor ethylene molecules as a function of distance.

Supplementary Fig. 40. Structures showing ethylene adsorption within the micropores of MFI structures. Siliceous MFI (S1) along (a) Y and (b) X directions, silanol-modified siliceous MFI (S1-OH) along (c) Y and (d) X directions.

Supplementary Fig. 41. Structures showing toluene adsorption within the micropores of MFI structures. Siliceous MFI (S1) along (a) Y and (b) X directions, silanol-modified siliceous MFI (S1-OH) along (c) Y and (d) X directions.

Supplementary Fig. 42. Mean square displacement (MSD) of ethylene molecules diffusing in the S1-OH zeolite.

Supplementary Fig. 43. Data characterizing catalytic performances of the Rh/SiO_2 and Rh/S1-OH catalysts in the hydroformylation of styrene and 2,4,6-trimethylstyrene.

Supplementary Fig. 44. Data showing the catalytic performances of various catalysts in the hydroformylation of styrene.

Supplementary Table 1. Data showing the Rh loadings on different catalysts.

Supplementary Table 2. TOF comparison of various catalysts in hydroformylation of olefins.

Supplementary Table 3. Data characterizing the performances of the Rh/S1-OH catalyst in the hydroformylation of various substrates.

Supplementary Table 4. Parameter of the force fields for zeolites and ethylene in theoretical simulation.

Supplementary Table 5. Adsorption energy for various molecule in S1 and S1-OH zeolites.

Supplementary Methods

Materials. All reagents were commercially obtained without purification. Tetraethyl orthosilicate (TEOS), tetrapropylammonium hydroxide (TPAOH, 40 *wt*%) and ammonia solution (25-28 *wt*%) were supplied by Shanghai Cairui Chemical Technology Co. Ltd. Rhodium chloride hydrate was obtained from Beijing HWRK Chem Co. Ltd. SiO_2, γ-Al_2O_3, styrene, 1-hexene, 1-octene, 4-methylphenylene, 4-chlorostyrene, alpha-methylstyrene, $Ga(NO_3)_3{\cdot}xH_2O$, and diethoxydimethylsilane (DEMS) were obtained from Aladdin Chemical Reagent Company. Commercial silicate-1 zeolite (denoted as S1-OH in this work) was obtained from Nankai University Catalyst Co. Ltd.

Synthesis of S-1 zeolite. 3 mL of TPAOH (40 *wt*%) and 3.5 g of TEOS were mixed in 11.28 g of water, stirring at room temperature for 6 h. Then, the mixture was transferred into an autoclave and hydrothermally treated at 180 °C for 72 h. After filtrating, washing with water, and calcining in the air at 550 °C for 4 h to remove the organic template, the siliceous MFI zeolite was finally obtained. By NMR analysis, the sample has silanol groups that mostly existed on the zeolite external surface. Therefore, it was denoted as S-1 for distinguishing from S1-OH.

Synthesis of S1-OH-x zeolite (x=5, 10, 20). The hydroxyl group modified S-1 zeolite was synthesized using DEMS and TEOS as silica sources, and the final products were denoted as S1-OH-*x*, where *x%* was the molar percentage of DEMS to the total amount of silica in the starting gels. As a typical run for the synthesis of S1-OH-5, 80 mL of ethanol was added to 100 mL of a water solution containing 6 mL of aqueous ammonia under stirring. Then, 3.90 g of TEOS and 0.15 g of DEMS were added and stirred at room temperature for another 8 h. After distilling under a vacuum to remove the water and ethanol and drying at 100 °C for 12 h, the solid powder of amorphous silica modified with methyl groups (SiO_2-Me) was obtained. The S-1-OH-5 zeolite was synthesized by solvent-free crystallization of SiO_2-Me in the presence of TPAOH. After grinding 0.5 g of TPAOH and 0.6 g of SiO_2-Me at room temperature for 10 min, the mixture was transferred into an autoclave for crystallization at 180 °C for 72 h to give the methyl-modified S-1 zeolite. After calcination at 550 °C in air for 4 h, the organic template was removed, and the methyl groups were transformed into hydroxyl groups, which were denoted as S1-OH-5. Furthermore, S1-OH-10 and S1-OH-20 samples were synthesized from the same procedures except using 3.75 g of TEOS with 0.30 g of DEMS, and 3.33 g of TEOS with 0.60 g of DEMS as the silica sources, respectively.

Synthesis of Rh/S1-OH zeolite. Rh/S1-OH was synthesized by an impregnation method. Typically, 1 g of S1-OH zeolite was pre-dehydrated at 200 °C and mixed with 2 mL of $RhCl_3{\cdot}3H_2O$ solution (Rh concentration at 3.9 μmol/mL). Then, the mixture was ultrasonically treated at room temperature for 1 h, followed by grinding the mixture at 60-70 °C to remove the water. The obtained powder was dried at 100 °C for 6 h and calcined at 400 °C for 3 h in air, and reduced at 400°C for 2 h in flowing hydrogen (linear heating to 400 °C, holding for 2 h, 10 *vol*% H_2 in Ar, flow at 60 mL/min) for obtaining the Rh/S1-OH catalyst. The accurate Rh loading in the final Rh/S1-OH zeolite was analyzed by ICP at 0.06 wt%, which is comparable to that in the starting mixture (Rh fraction to the zeolite was 0.08 wt%), suggesting that most of the Rh species have been successfully loaded on the zeolite support.

Synthesis of Rh/S-1, Rh/TiO_2, Rh/Al_2O_3, Rh/SiO_2, and Rh/CeO_2. These catalysts were synthesized from the same procedures of Rh/S1-OH except different solid carriers that were used for supporting Rh nanoparticles.

Synthesis of siliceous zeolite with blocked micropores. This material was synthesized from the same procedures with S1-OH-5 zeolite without the calcination at 550 °C. The Rh nanoparticles were loaded by an impregnation method according to the aforementioned procedures.

Synthesis of Rh/MFI-deGa-30 and Rh/MFI-deGa-60. As a typical run for synthesizing Ga-MFI zeolite with Si/Ga ratio at 30, 0.134 g of $Ga(NO_3)_3{\cdot}xH_2O$, 3 mL of TPAOH (40 *wt*%), and 3.27 g of TEOS were mixed in 11.28 g of water and stirred at room temperature for 6 h. Then, the mixture was transferred into an autoclave and hydrothermally treated at 180 °C for 72 h. After filtrating, washing with water, and calcining in air at 550 °C for 4 h to remove the organic template, the Ga-MFI zeolite was finally obtained. The degallation was performed for obtaining siliceous zeolite with abundant silanol nests[1]. 1 g of Ga-MFI zeolite was mixed in 100 mL of nitric acid (2 M) and then stirred at 85°C for 6 h. After filtrating and washing with water, the siliceous zeolite with removed gallium species was obtained, which were denoted as MFI-deGa-30. MFI-deGa-60 zeolite was synthesized from the same procedures except using 0.067 g of $Ga(NO_3)_3{\cdot}xH_2O$ in the starting gel. Rh/MFI-deGa-30 and Rh/MFI-deGa-60 were prepared by loading Rh nanoparticles on these zeolites according to the same procedures in preparing Rh/S1-OH.

Catalyst characterizations.

X-ray diffraction (XRD) data were collected on a Rigaku D/MAX 2550 diffractometer with Cu Kα (λ=1.5418 Å). The step size was 0.02°, and the scanning speed was 20°/min. The amount of Rh element was determined by inductively coupled plasma (ICP) analysis (Perkin-Elmer 3300DV). Nitrogen sorption isotherms were measured using a Micromeritics ASAP2020 system. SEM experiments were performed using Hitachi SU-1510 electron microscopes. TEM was performed on a JEM-2100F electron microscopy (JEOL, Japan) with an acceleration voltage of 200 kV. In the TEM, the sample was loaded on a Cu mesh with carbon film. X-ray photoelectron spectroscopy (XPS) was used to test the Rh *3d* binding energy values of different Rh samples on AXIS Supra (Kratos). The binding energy (BE) values were calibrated against the C1s signal (BE=284.8 eV) of contaminant carbon. Mass spectra of the effluent gases introduced into a flow system or produced by reaction with the sample were measured on a mass spectrum (SRD200M, TILON GRP TECHNOLOGY LIMITED) instrument connected to a reaction tube, whose temperature was controlled by an oven. In the C_2H_4-TPD, the catalyst sample (100 mg) was pretreated at 400°C in 10% H_2/He flow (30 mL/min) for 1h, followed by the adsorption of C_2H_4 at 30 °C for 1h. After saturation, the sample was purged using the He flow for 1 h. Then, desorption of C_2H_4 was carried out from 100 to 650 °C with a heating rate of 10 °C/min. The liquid NMR spectra were recorded with a Bruker Avance-400 spectrometer. Chemical shifts are expressed in ppm downfield from TMS at $\delta = 0$ ppm. ^{13}C (100.5 MHz) cross-polarization magic-angle spinning (CP-MAS), and ^{31}P (161.8 MHz). MAS solid-state NMR experiments were recorded on a Varian infinity plus 400 spectrometers equipped with a magic-angle spin probe in a 4-mm ZrO_2 rotor. The ^{31}P NMR chemical shifts were referenced to the $(NH_4)_2HPO_4$.

FTIR spectra were recorded on a Perkin-Elmer spectrometer (Spectrum TM GX). All spectra were collected with an average of 64 scans at 2 cm^{-1} resolutions. Prior to the measurement, the samples were pretreated in flowing hydrogen (10 *vol*% H_2 in Ar) in an *in situ* cell at 400 °C for 1 h. After that, the sample cell was vacuumed to <10^{-3} Pa to remove the physically adsorbed species. Then the cell was cooled down to 30 °C to collect FTIR spectrum for observing the silanol signals. The olefin-adsorption FTIR spectra were collected by pulsing the olefin substrates to the sample cell. The olefins were pulsed multiple times to collect the spectra under different partial pressures. After each pulse, the sample was equilibrated for 20 min prior to collecting the FTIR spectrum.

The solid-state magic-angle-spinning (MAS) NMR experiments were performed on a Bruker AVANCE-III 500 MHz spectrometer operating at a Larmor frequency of 500.57 and 99.44 MHz for ^{1}H and ^{29}Si nucleus, respectively. The ^{1}H NMR spectra were recorded using a 4 mm triple-resonance MAS probe at a spinning rate of 12 kHz. A single-pulse sequence with a $\pi/2$ pulse length of 4.0 μs and a recycle delay of 5 s was used for the one-dimensional (1D) ^{1}H MAS NMR experiments. For the two-dimensional (2D) ^{1}H-^{1}H double quantum (DQ) MAS NMR measurements, DQ coherences were excited and reconverted with a POST-C7 pulse sequence. The increment interval in the indirect

dimension was 80 μs. Typically, 128 scans were acquired for each t_1 increment, and two-dimensional data sets consisted of 128 t_1 × 512 t_2 [2]. ^{29}Si MAS NMR spectra with high power proton decoupling were recorded using a π/2 pulse of 3.9 μs and a recycle delay of 60 s on a 7 mm MAS probe with a spinning rate of 5 kHz. For the ^{1}H-^{29}Si CP MAS NMR measurements, the Hartmann-Hahn condition was achieved with a contact time of 4 ms and a recycle delay of 3 s. The chemical shifts of ^{1}H and ^{29}Si nucleus were externally referenced to adamantane (1.91 ppm) and kaolinite (-91.5 ppm). Prior to ^{1}H NMR experiments, each sample was placed in a glass tube and connected to a vacuum line for dehydration. The temperature was gradually increased at a rate of 1 K/min, and the sample was kept at a final temperature of 673 K under a pressure below 10^{-3} Pa over a period of 10 h and was then flame-sealed. Prior to the NMR experiments, the sealed sample was transferred into a ZrO_2 rotor (tightly sealed by a Kel-F cap) under a dry nitrogen atmosphere in a glove box.

Catalytic tests in hydroformylation.

The hydroformylation was performed in a high-pressure stainless-steel autoclave containing a Teflon liner vessel with a total volume of 50 mL. As a typical run for the hydroformylation of styrene, 30 mg of catalyst and 2.5 mmol of styrene were mixed in 5 mL of toluene solvent in the autoclave. Then, the air in the autoclave was removed by nitrogen, followed by pumping of syngas with a molar ratio of CO to H_2 at 1 (molar ratio of CO/H_2/Ar at 45/45/10). The syngas was pumped three times to remove the nitrogen in the autoclave, and maintained a high pressure at 3.0 MPa, followed by heating to start the reaction. It will cost about 30 min to reach the desired reaction temperature, and this time was involved in the reaction time described in the reaction conditions. The temperature was measured by a thermocouple inserted into the internal autoclave. After the reaction, the autoclave was rapidly cooled in a water-ice mixture. The residual CO in the reactor was detected by gas chromatography with a thermal conductivity detector. After pressure releasing in the autoclave, and 1-butanol was added to the liquor as an internal standard. Then, the catalyst was separated, and the liquor was analyzed by gas chromatography with a flame ionization detector and FFAP (free fatty acid polyester) column.

As a typical run for the hydroformylation of ethylene, the 3.0 MPa of the gas containing 0.1 MPa of methane, 0.5 MPa of ethylene, and 2.4 MPa of syngas (molar ratio of CO/H_2/Ar at 45/45/10) were fed to the autoclave containing toluene solvent and catalyst. After the reaction, the ethylene conversion was obtained by analyzing the gaseous composition using gas chromatography with a flame ionization detector and Al_2O_3 column. Methane in the feed was used as internal standard. A very slight amount of ethylene (about 0.1% of the ethylene in the feed) existed in the toluene solvent, which was not involved in calculating the ethylene conversion. The liquor was analyzed by gas chromatography with a flame ionization detector and FFAP column using 1-butanol as an internal standard for obtaining the propanal yield.

Turnover frequency (TOF) values were measured in a high-pressure stainless-steel autoclave containing a Teflon liner vessel with a total volume of 50 mL. 15 mg of catalyst and 20 mmol of styrene were mixed in 5 mL of toluene solvent in the autoclave. Air in the autoclave was removed by nitrogen, followed by pumping syngas with a molar ratio of CO to H_2 at 1.0 (molar ratio of CO/H_2/Ar at 45/45/10) three times to remove the nitrogen in the autoclave. The pressure was maintained at a desired pressure at 3.0 MPa, followed by heating to 110 °C to start the reaction. The temperature was mintored by a thermocouple inserted into the internal autoclave. After reaction for different periods, the phenylpropyl aldehyde yields were analyzed (styrene conversation controlled to be lower than 20%) for calculating the reaction rates. According to the Rh dispersion, the TOF was calculated at 50,306 h^{-1}, which was described as 50,000 h^{-1} considering the significant digit issue.

Theoretical simulation

Model A (Supplementary Fig. 36) simulates the homogeneous system and S1-OH zeolite surface. The initial

framework structure of pure silicon MFI was taken from the IZA database, and one of the Si (T12) atoms was deleted, as well as hydroxyl groups were added to the unsaturated atoms. Then 3 × 3 × 4 supercells were selected, and the S1-OH zeolite surface was obtained by disrupting the periodicity of the bulk zeolite, as well as a 60 Å vacuum layer was added also along [010]. It should be noted that the hydroxyl groups were added to the unsaturated atoms over the external surface. The number of ethylene was 50, 100 and 200.

Model B (Supplementary Fig. 38) simulates the ethylene molecules in a periodic structure. The box was chosen as 120.5 × 120.0 × 105.1 $Å^3$ and 60.3 × 59.2 × 52.6 $Å^3$ for (Supplementary Fig. 38a) gas and S1-OH zeolite (Supplementary Fig. 38b). The number of ethylene was 89, which was consistent with the density of model A after equilibration.

DFT simulation

All geometry optimizations were performed according to the plane-wave-based periodic DFT method as implemented in the Vienna Ab Initio Simulation Package [3,4]. The electron-ion interaction was described with the projector augmented wave method [5,6]. The electron exchange and correlation energies were treated within the generalized gradient approximation in the Perdew-Burke-Ernzerhof functional (GGA-PBE) [7]. The energy cutoff of the plane-wave basis was set up to 400 eV. Electron smearing was used via the Methfessel-Paxton technique to speed up the convergence of the metallic systems with a width of σ = 0.2 eV and the 1 × 1 × 1 gamma-point sampling was used for geometry optimization.

The adsorption energy was calculated according to $E_{ads} = E_{x/slab} – [E_{slab} + E_x]$, where $E_{x/slab}$ was the total energy of the slab with adsorbates in its equilibrium geometry, E_{salb} was the total energy of the bare slab, and E_x was the total energy of the free adsorbates in the gas phase. Therefore, the more negative for the value of E_{ads}, the stronger for the adsorption.

Molecular dynamics (MD) simulation

MD simulations were performed in the canonical ensemble (NVT), where the number of particles (N), volume (V), and temperature (T) were kept constant. The simulated temperature was 110 °C and controlled by a Nosé-Hoover thermostat with a coupling time constant of 0.1 ps. The leapfrog Verlet algorithm was used to integrate the Newton's equations of motion with a time step of 1 fs. The TraPPE-UA [9] and revised TraPPE-Zeo [8-11] force field was used for hydrocarbon and zeolites (Supplementary Table 4), respectively. All the Lennard-Jones cross-interaction parameters were determined by Lorentz–Berthelot mixing rules. Each MD simulation was performed by 1.5 × 10^7 steps. The cutoff radius was 14 Å, and the trajectories were recorded every 1000 steps. 5 independent MD simulations were carried out for better statistics. All MD simulation were performed in the DL_POLY 2.0 code [12].

Mean square displacement and diffusion coefficient

The mean square displacement (MSD) of ethylene was defined as equation in the following [13]:

$$MSD(\tau) = \frac{1}{N_m}\sum_{i}^{N_m}\frac{1}{N_\tau}\sum_{t_0}^{N_t}\left[r_i(t_0+\tau) - r_i(t_0)\right]^2 \quad (1)$$

where N_m was the number of gas molecules, N_τ was the number of time origins used in calculating the average, and r_i was the coordinate of the i-th molecule. In addition, the slope of the MSD as a function of time determined the self-diffusion coefficient, D_s, which was defined according to the so-called Einstein relation.

$$MSD(\tau) = 2nD_s\tau + b \quad (2)$$

where n was the dimension of zeolites. In this work, n = 1 for the diffusion along with straight channel of S1-OH.

The reported MSD and D_s values were calculated as the average of 5 independent MD trajectories.

Supplementary Figures

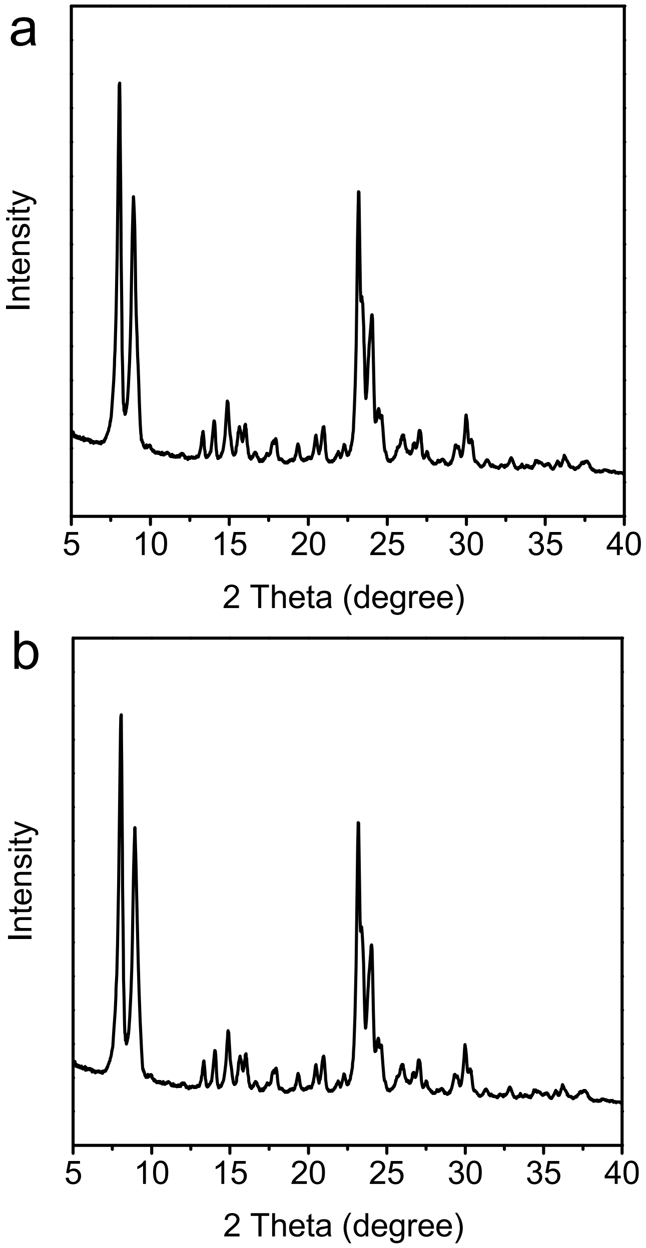

Supplementary Fig. 1. XRD patterns of the (a) S1-OH and (b) Rh/S1-OH. Both samples exhibited typical peaks assigned to MFI zeolite structure.

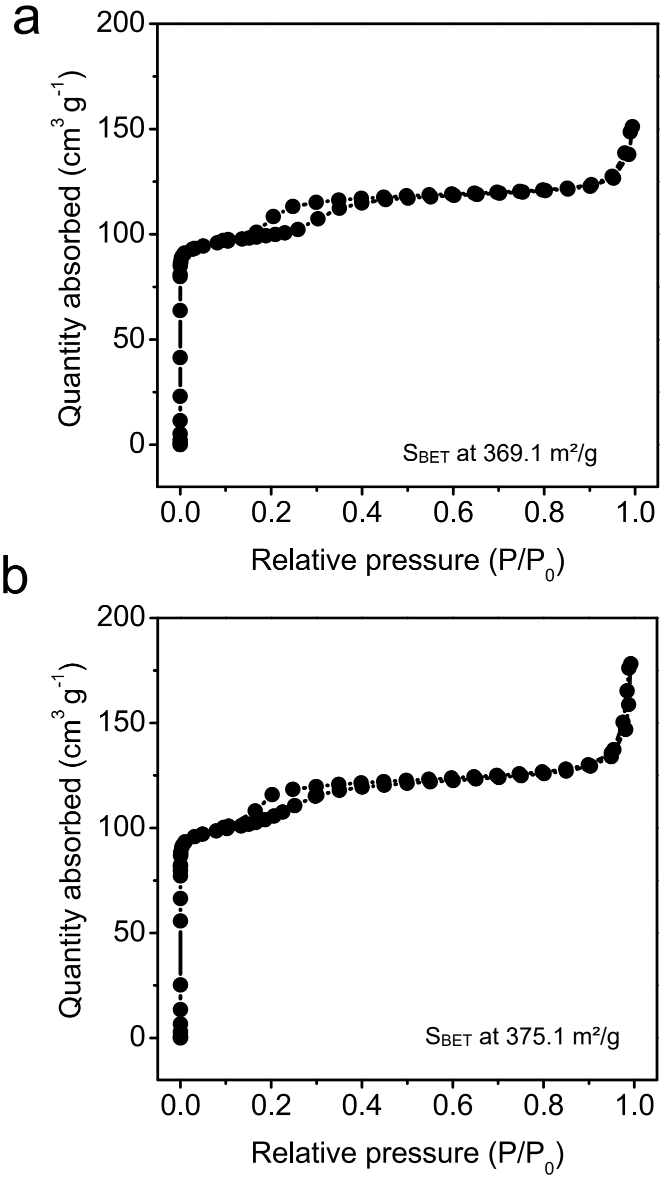

Supplementary Fig. 2. N_2 sorption isotherms of the (a) S1-OH and (b) Rh/S1-OH samples.

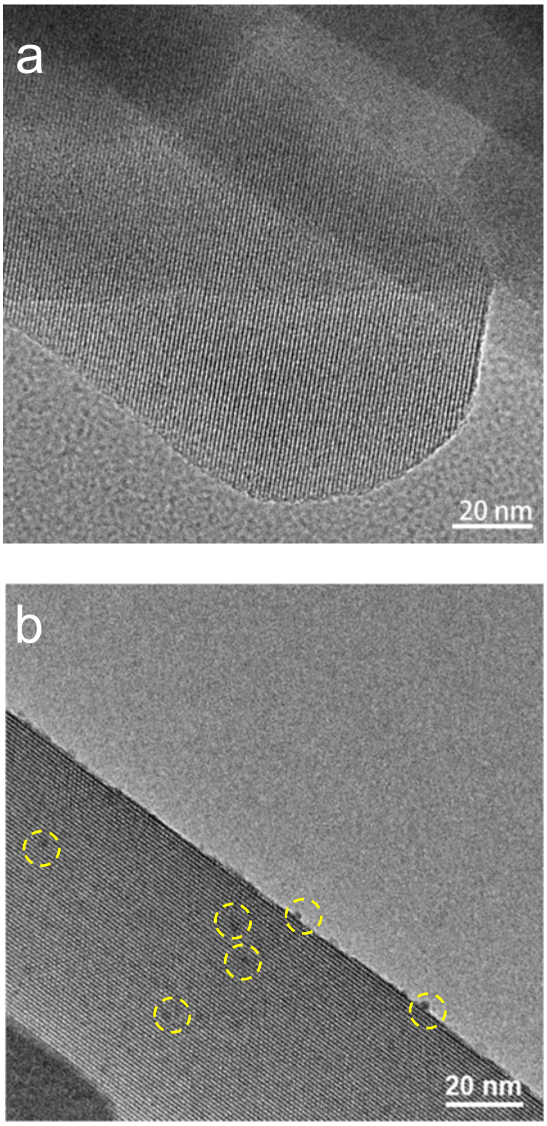

Supplementary Fig. 3. TEM images of the (a) S1-OH and (b) Rh/S1-OH samples. The yellow circles highlighted parts of the Rh nanoparticles.

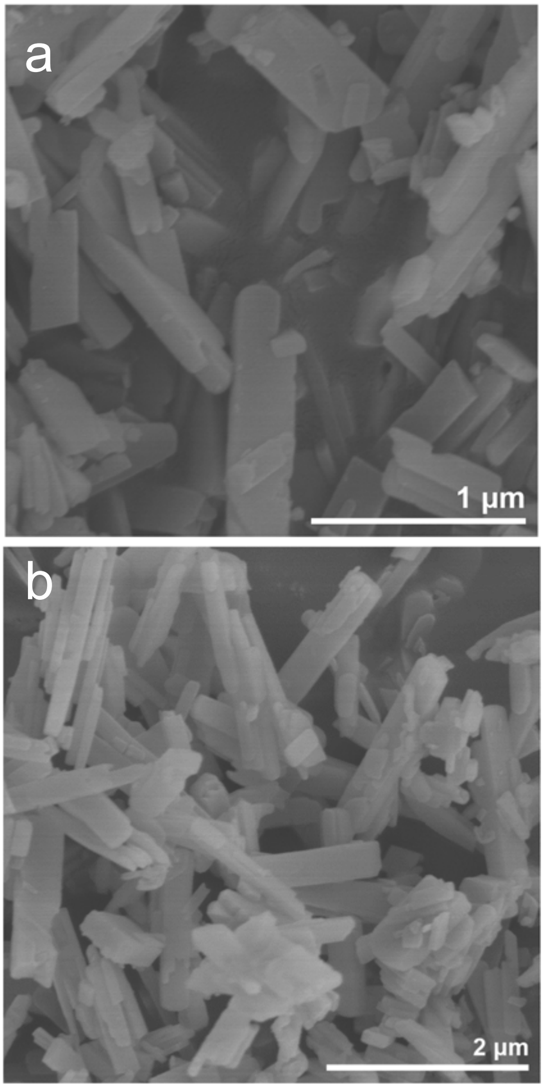

Supplementary Fig. 4. SEM images of the (a) S1-OH and (b) Rh/S1-OH samples. The thickness of the zeolite crystals was about 230 nm.

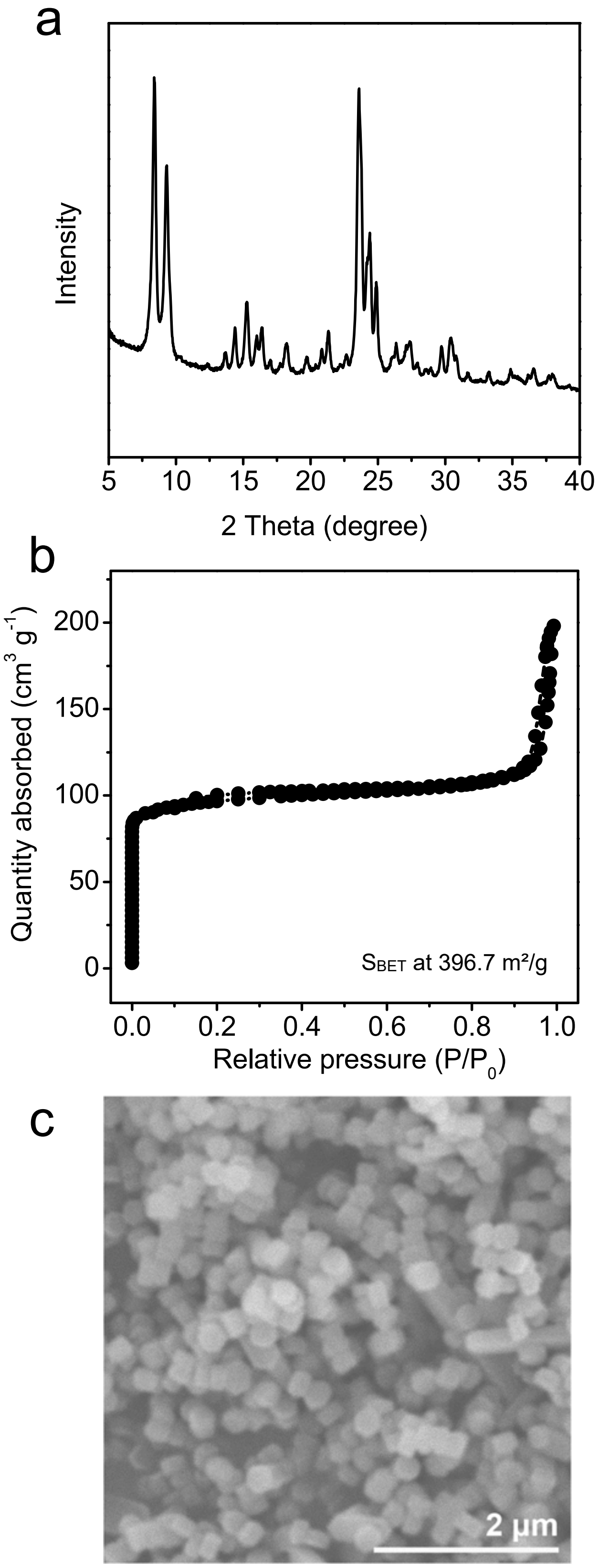

Supplementary Fig. 5. (a) XRD pattern, (b) N_2 sorption isotherms, and (c) SEM image of the S-1 sample. The average diameter of the zeolite crystals was about 300 nm.

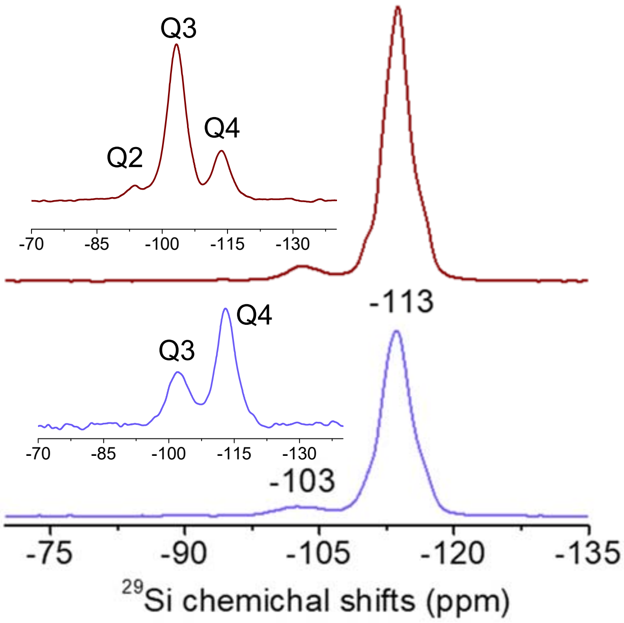

Supplementary Fig. 6. ^{29}Si MAS NMR spectra of S1-OH and S-1 zeolites. Inset, ^{29}Si CP/MAS NMR spectra of the two samples.

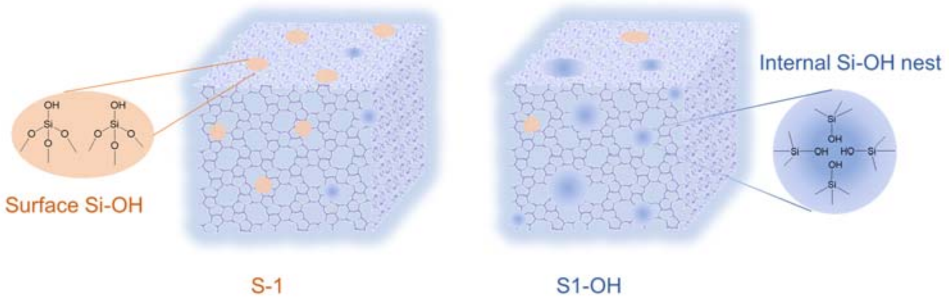

Supplementary Fig. 7. Scheme showing the structure of different silanols in zeolites.

a
b
c

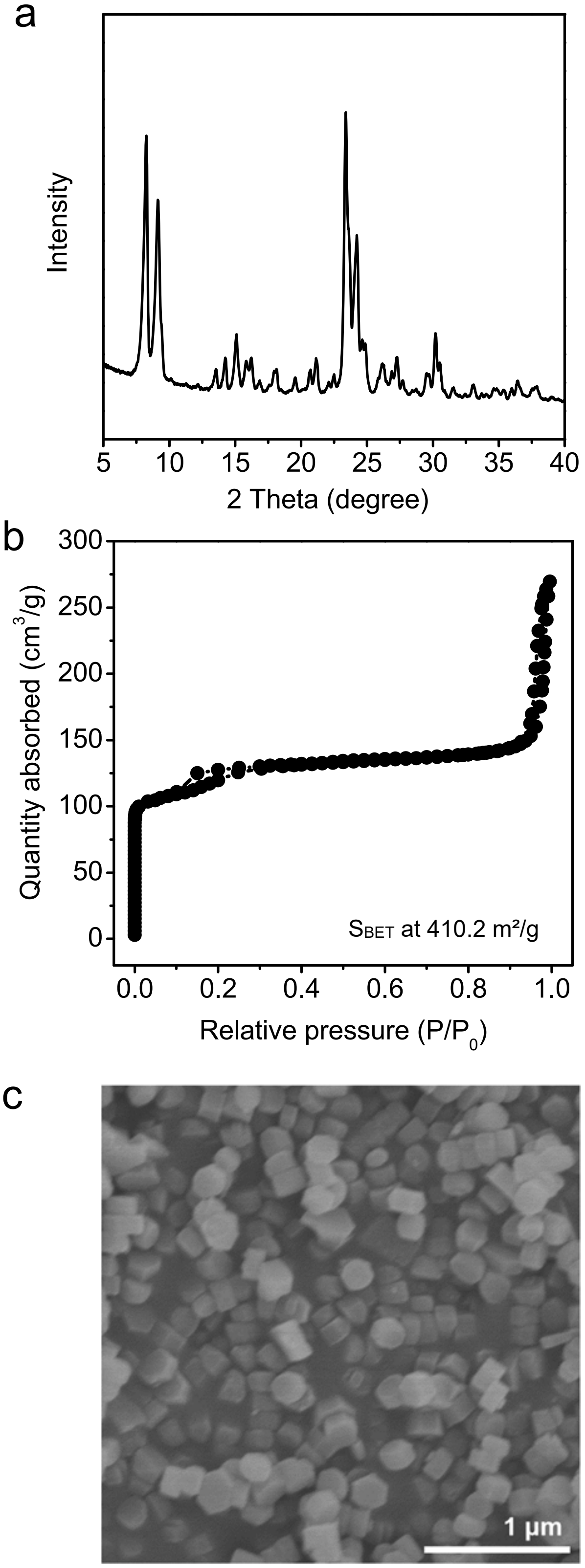

Supplementary Fig. 8. (a) XRD pattern, (B) N_2 sorption isotherms, and (c) SEM image of the Rh/S-1 sample.

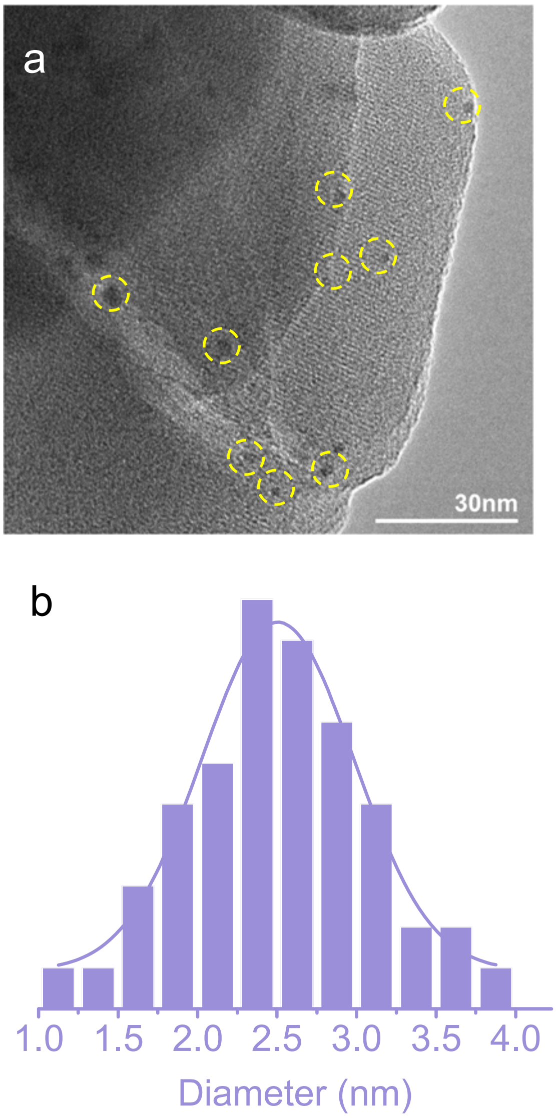

Supplementary Fig. 9. (a) TEM image and (b) Rh nanoparticle size distribution of the Rh/S-1 sample.

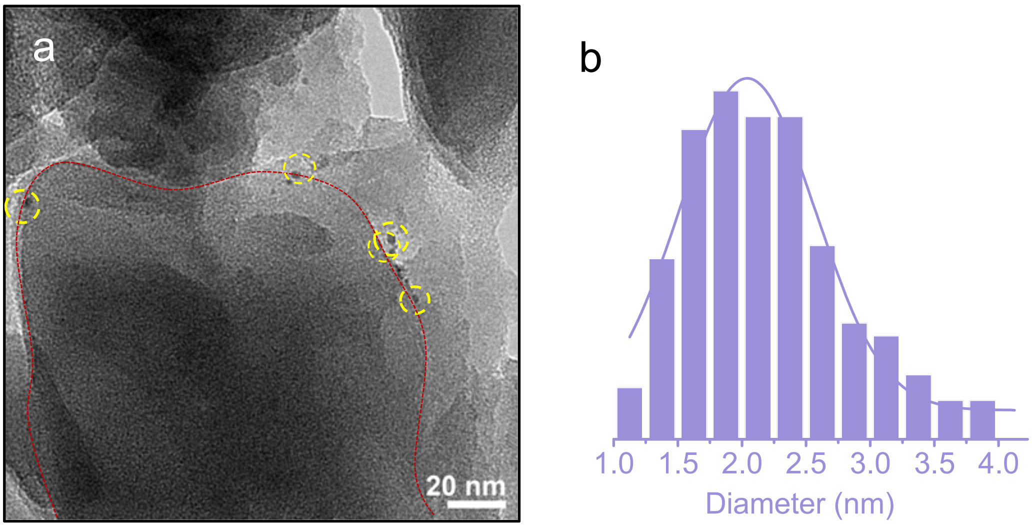

Supplementary Fig. 10. (a) Tomographic section TEM image and (b) Rh nanoparticle size distribution of the Rh/S1-OH samples. Zeolite crystal was outlined with red line. The yellow circles highlight part of the Rh nanoparticles.

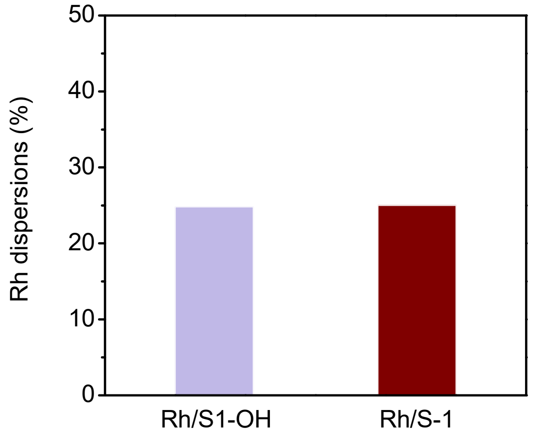

Supplementary Fig. 11. Rh dispersions of the (a) Rh/S1-OH and (b) Rh/S-1 samples.

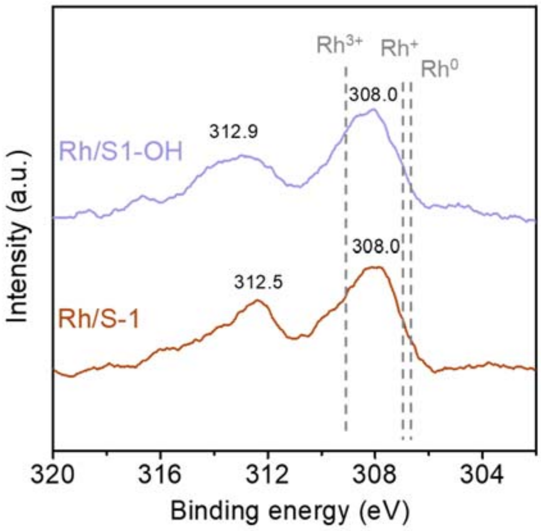

Supplementary Fig. 12. Rh *3d* XPS spectra of the Rh/S1-OH and Rh/S-1 samples.

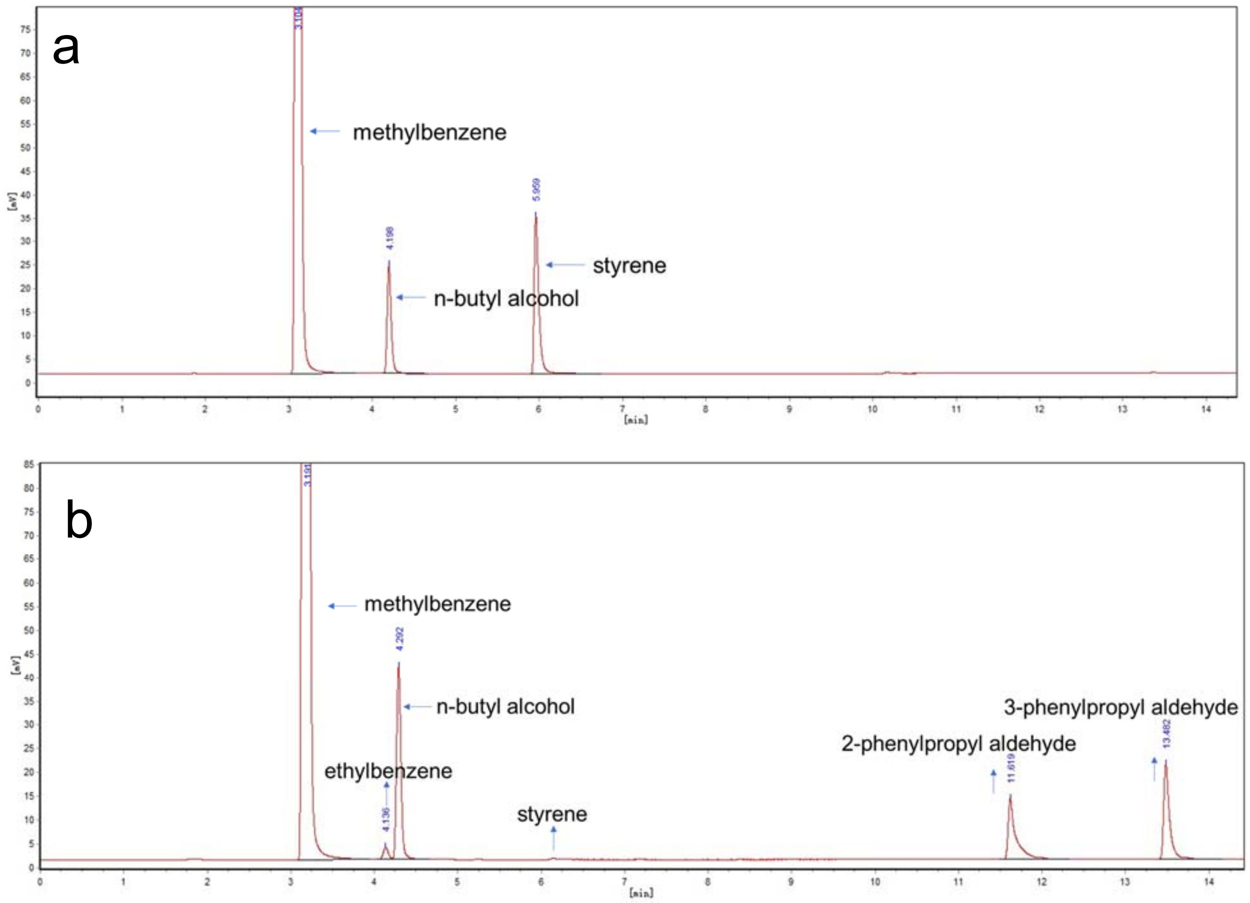

Supplementary Fig. 13. GC profiles analyzing the products from (a) S1-OH and (b) Rh/S1-OH catalyzed styrene hydroformylation, corresponding to the data in Figure 2a.

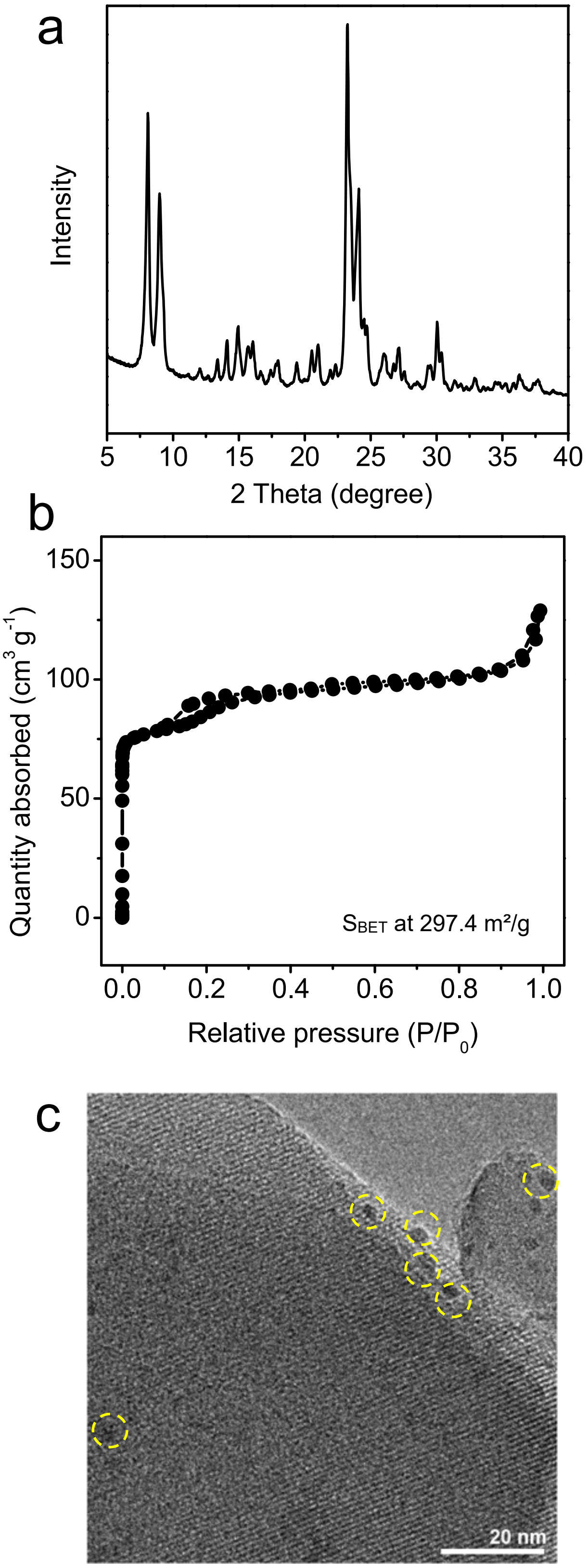

Supplementary Fig. 14. (a) XRD pattern, (b) N_2 sorption isotherms, and (c) TEM image of the Rh/ZSM-5 catalyst. The yellow circles highlighted the Rh nanoparticles.

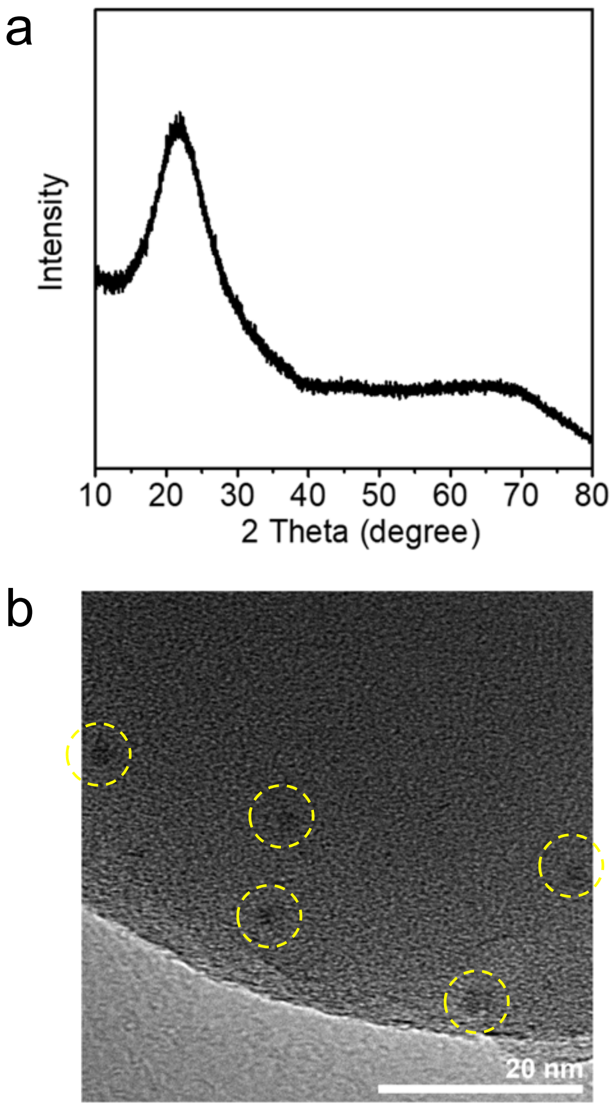

Supplementary Fig. 15. (a) XRD pattern and (b) TEM image of the Rh/SiO_2 catalyst. The yellow circles highlighted the Rh nanoparticles.

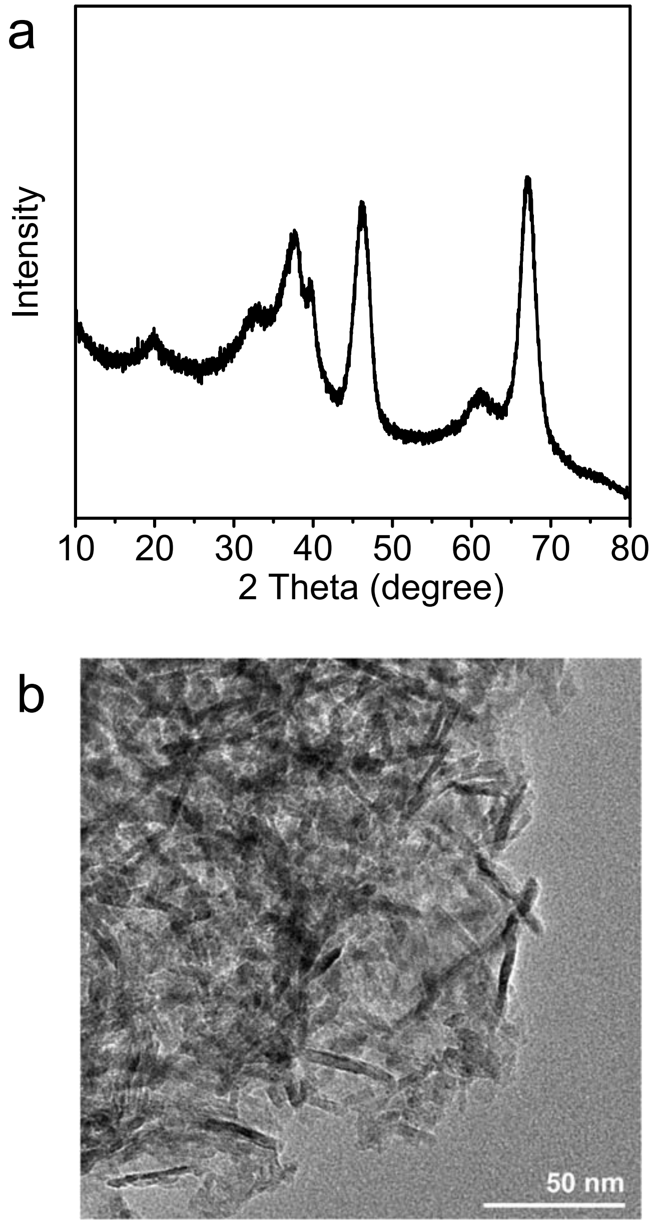

Supplementary Fig. 16. (a) XRD pattern and (b) TEM image of the Rh/γ-Al_2O_3 catalyst.

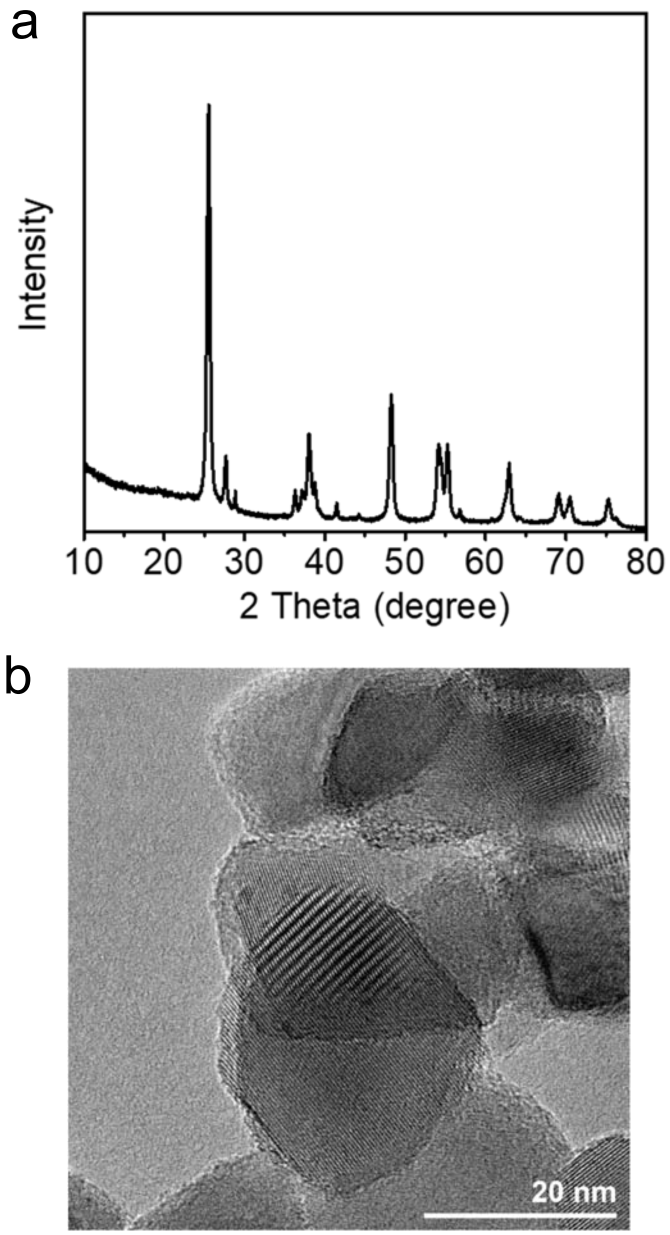

Supplementary Fig. 17. (a) XRD pattern and (b) TEM image of the Rh/TiO_2 catalyst.

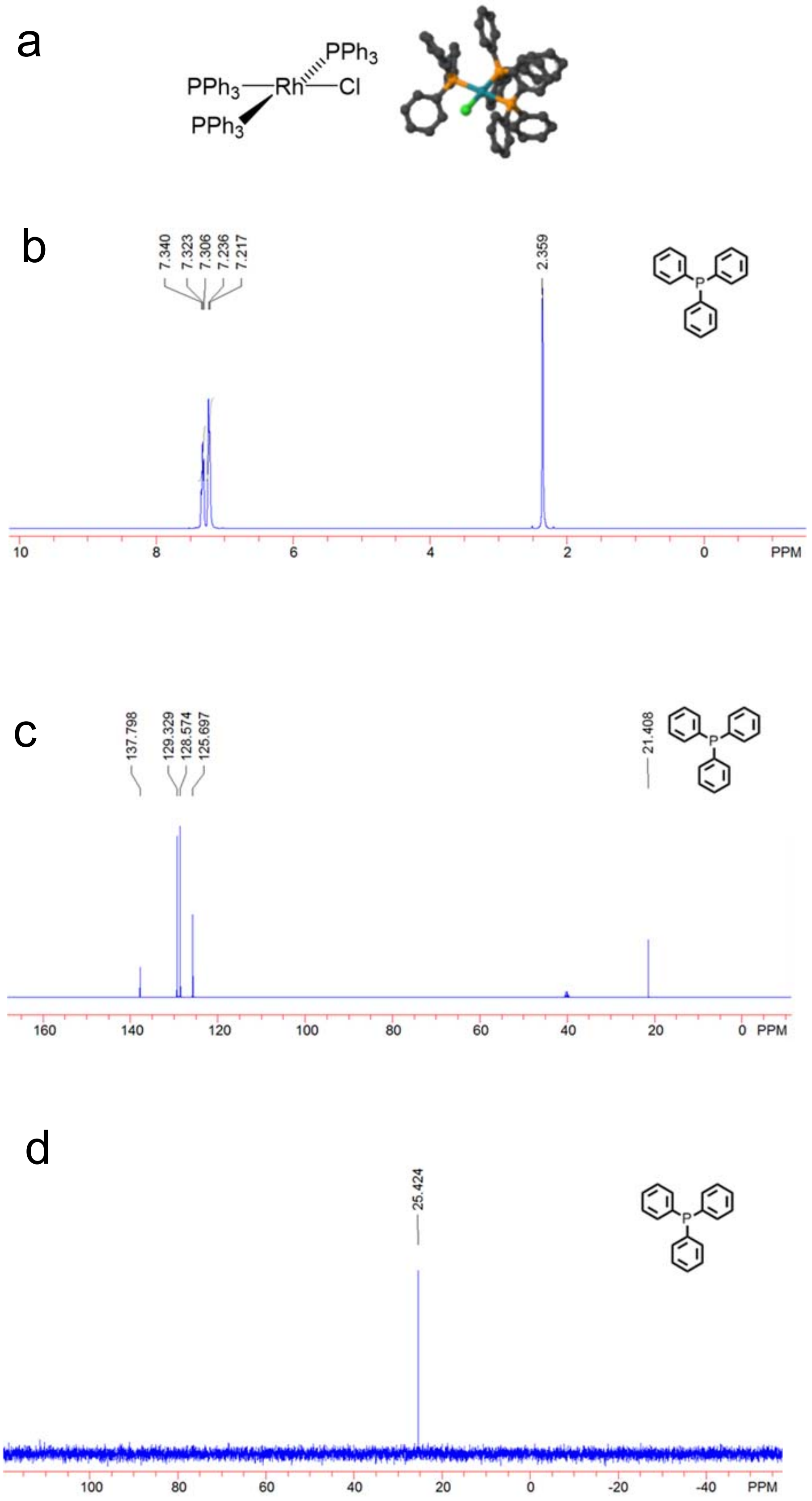

Supplementary Fig. 18. (a) Structural formula, (b) ^{1}H, (c) ^{13}C, and (d) ^{31}P NMR spectra of Wilkinson's catalyst.

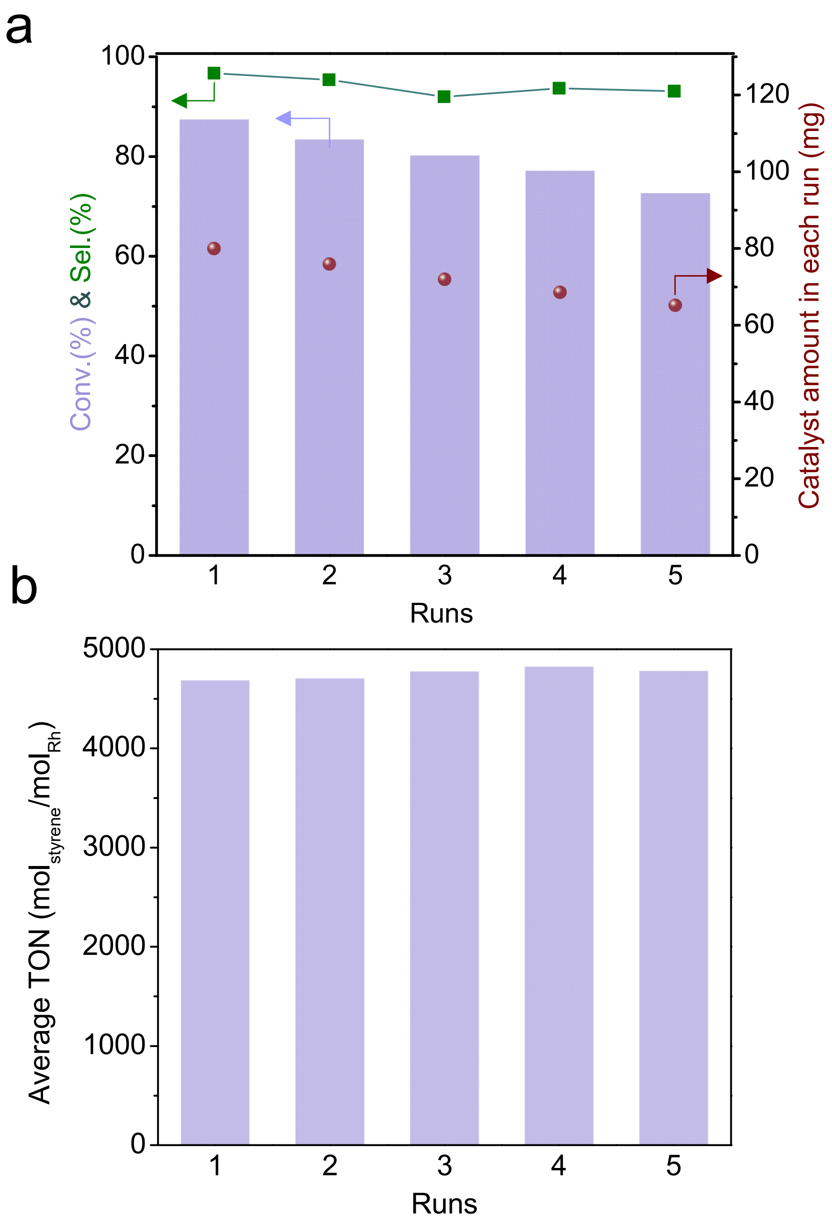

Supplementary Fig. 19. (a) Data characterizing the performances of the Rh/S1-OH in the recycle tests for styrene hydroformylation. (b) Reaction rate of the Rh/S1-OH in the recycle tests for styrene hydroformylation. Reaction conditions: 3.0 MPa of syngas with a molar ratio of CO to H_2 at 1 (molar ratio of CO/H_2/Ar at 45/45/10), 80 mg of catalyst in the 1st run, 2.5 mmol of styrene, 5 mL of toluene as solvent, 1-butanol as an internal standard, 110 °C, and 2.5 h. The carbon balances were over 99.5% for all the tests.

Note: After each reaction run, the catalyst was separated and dried at 30 °C under vacuum for 4 h and reused in the next run. In each cycle, about 5 wt% of the catalyst was lost (physical loss during the operation, the Rh leaching was negligible as confirmed by the ICP analysis), and the amounts of used catalyst in each run were presented in the figure. The data on average reaction rate in each test showed the unchanged activity of Rh/S1-OH.

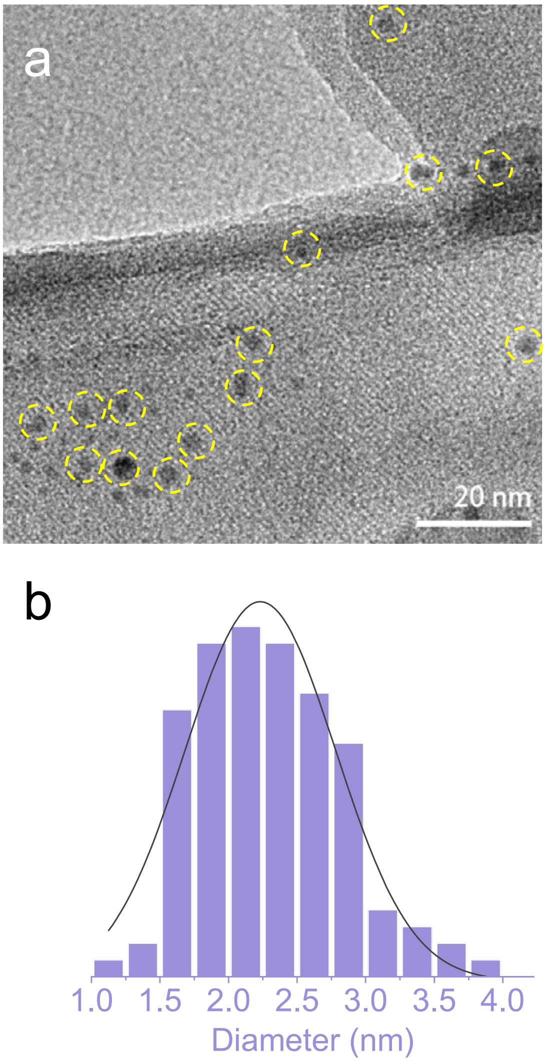

Supplementary Fig. 20. (a) TEM images and (b) Rh nanoparticle size distribution of the used Rh/S1-OH catalyst after the recycle tests. The yellow circles highlighted the parts of the Rh nanoparticles.

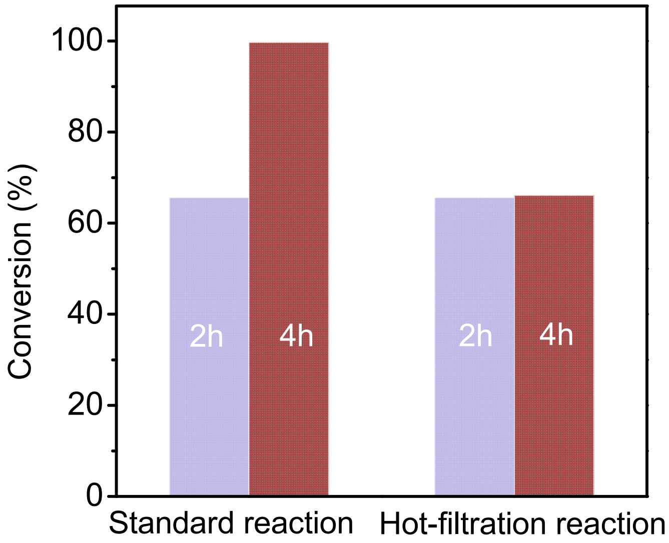

Supplementary Fig. 21. Data showing the styrene conversion in the standard reaction and hot-filtration reaction for 2 and 4 h. In the hot-filtration test, the catalyst was separated from the reaction liquor after 2 h, giving negligible styrene conversion in the reaction for additional 2 h.

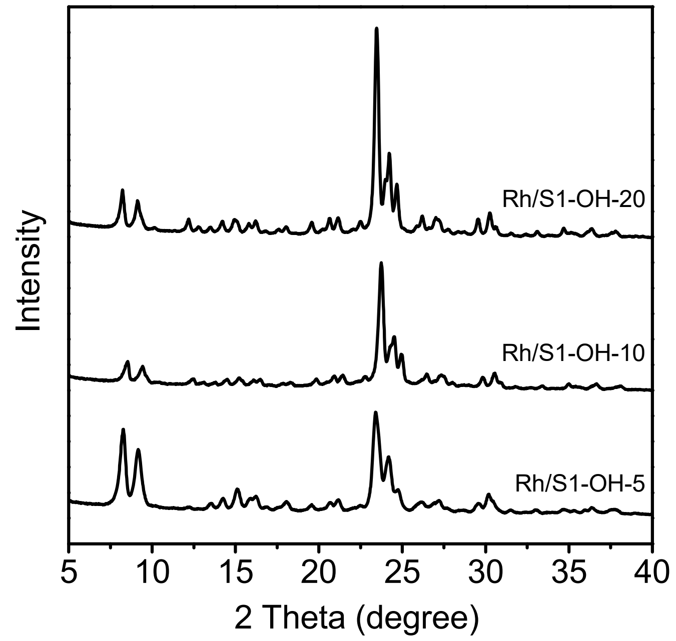

Supplementary Fig. 22. XRD patterns of the Rh/S1-OH-5, Rh/S1-OH-10, and Rh/S1-OH-20 samples.

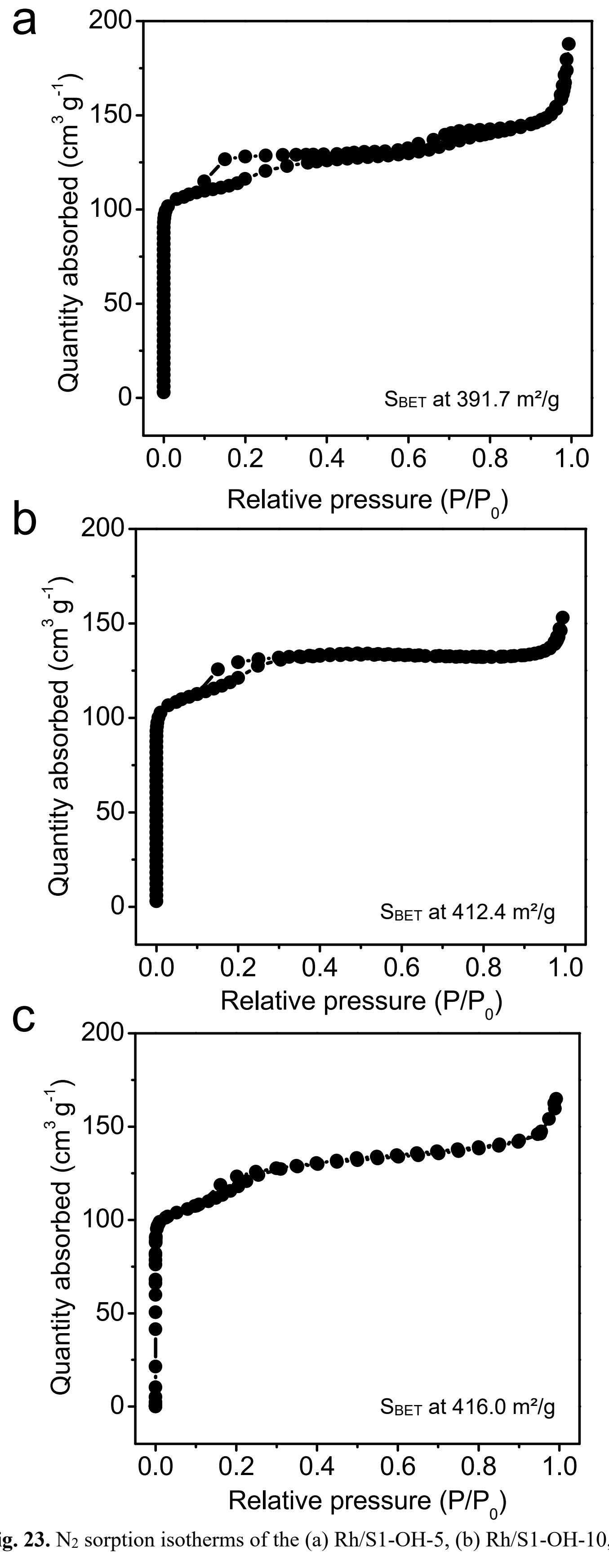

Supplementary Fig. 23. N_2 sorption isotherms of the (a) Rh/S1-OH-5, (b) Rh/S1-OH-10, and (c) Rh/S1-OH-20 samples.

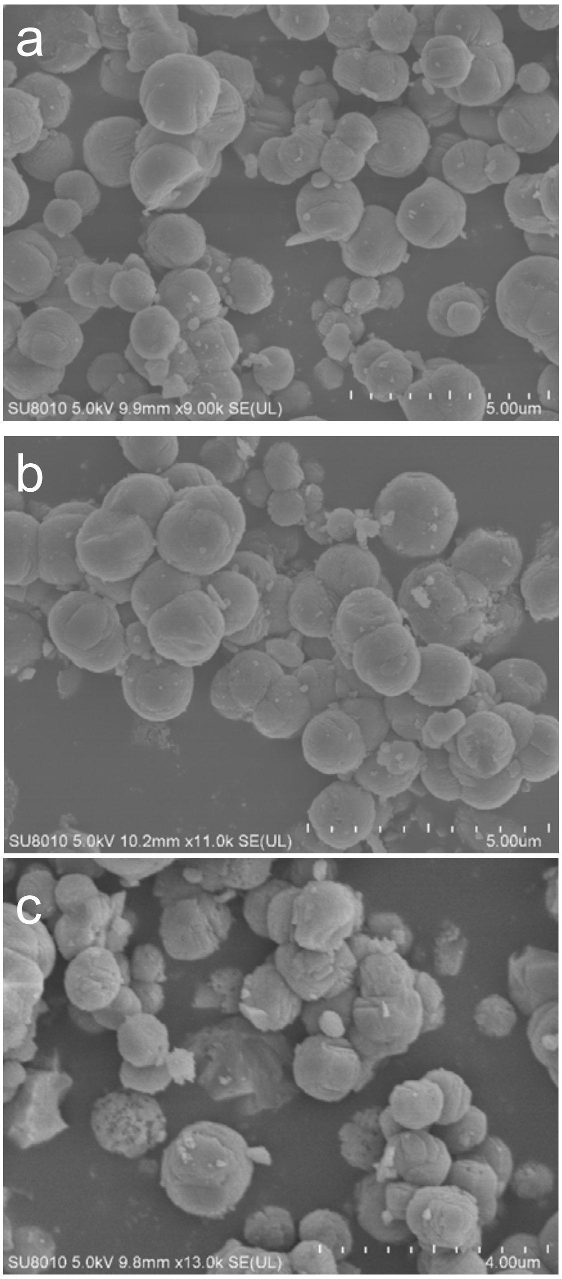

Supplementary Fig. 24. SEM images of the (a) Rh/S1-OH-5, (b) Rh/S1-OH-10, and (c) Rh/S1-OH-20 samples. All these samples exhibited very similar crystal sizes and morphology.

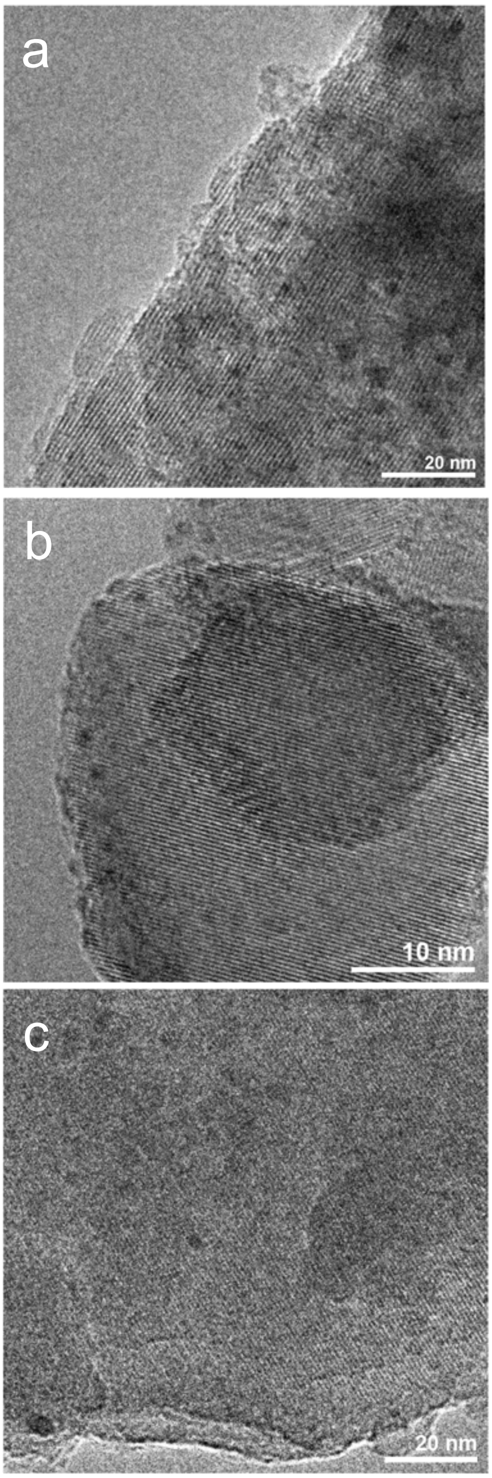

Supplementary Fig. 25. TEM images of the (a) Rh/S1-OH-5, (b) Rh/S1-OH-10, and (c) Rh/S1-OH-20. These catalysts exhibited similar Rh nanoparticle size distribution, as confirmed by the TEM characterizations, which would benefit the investigation of the zeolite silanols for the reaction to exclude the possible influence raised by the metal nanoparticle size difference.

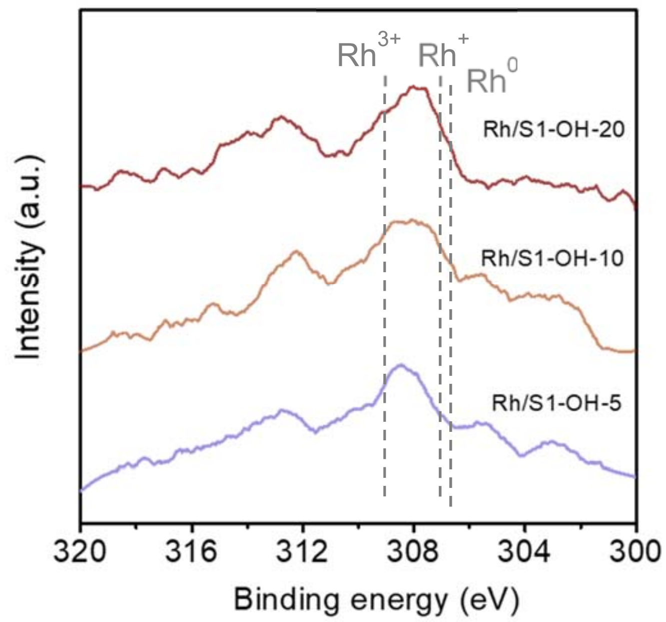

Supplementary Fig. 26. Rh *3d* XPS spectra of the Rh/S1-OH-5, Rh/S1-OH-10, and Rh/S1-OH-20 samples.

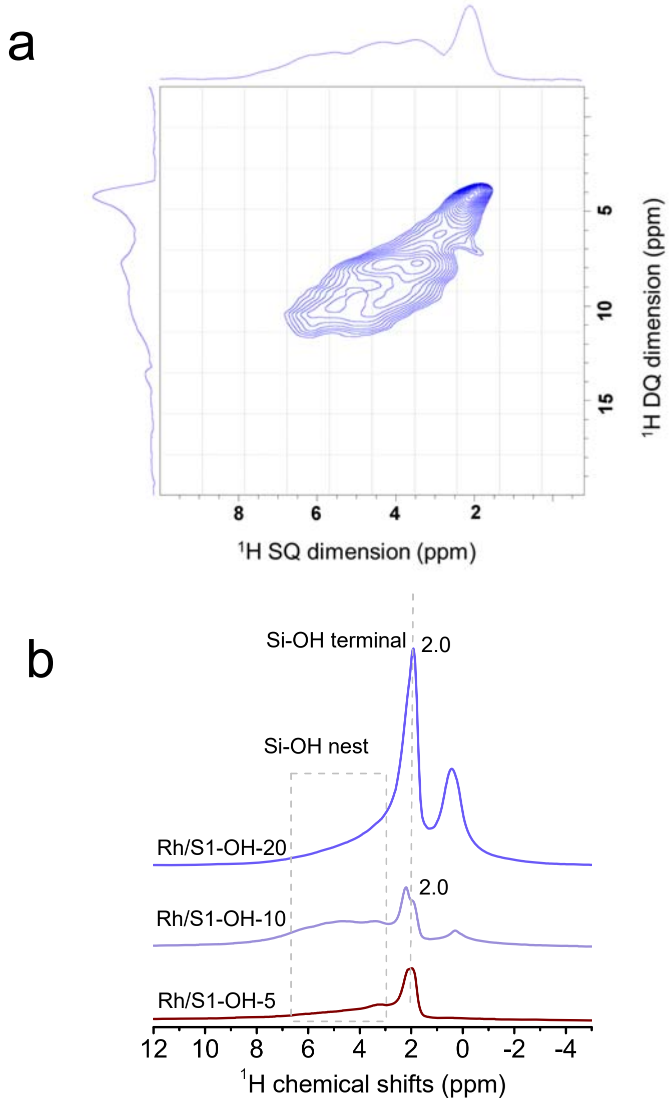

Supplementary Fig. 27. (a) 2D ^{1}H–^{1}H DQ MAS NMR of the Rh/S1-OH-5 sample. (b)^{1}H MAS NMR of the Rh/S1-OH-5, Rh/S1-OH-10, and Rh/S1-OH-20 samples.

Note: The 2D ^{1}H–^{1}H DQ MAS NMR spectra of Rh/S1-OH-5 showed obvious signals ranging from 3 to 8 ppm, suggesting the abundant silanol nests.

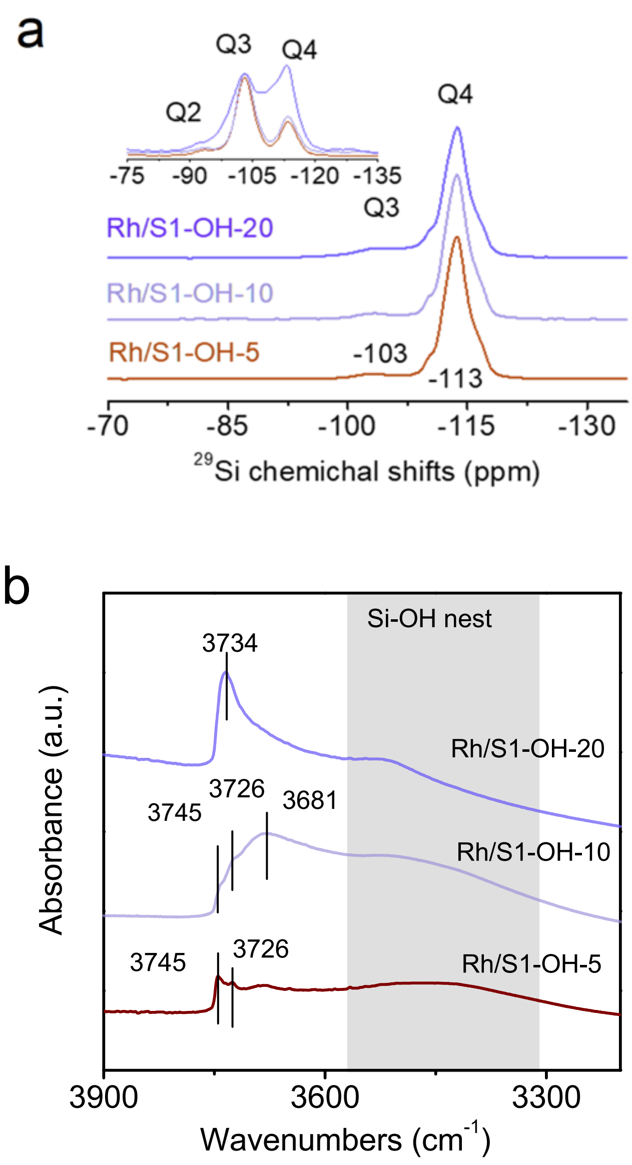

Supplementary Fig. 28. (a) ^{29}Si MAS NMR spectra of Rh/S1-OH-5, Rh/S1-OH-10, and Rh/S1-OH-20 samples. Inset in figure, ^{29}Si CP/MAS NMR spectra of the three samples. (b) FTIR spectra of the Rh/S1-OH-5, Rh/S1-OH-10, and Rh/S1-OH-20 samples.

Note: The FTIR spectrum of Rh/S1-OH-5 showed the bands at 3745, 3726, and 3681 cm^{-1}, which were assigned to the isolated silanol (3745 cm^{-1}) and the silanol nests (3726 and 3681 cm^{-1}, or silanol in the micropores). The broad signals at 3300-3500 cm^{-1} also supported the existence of abundant silanol nests. On the spectrum of Rh/S1-OH-10, the signals at 3726, 3681, and 3300-3500 cm^{-1} were obviously enhanced, confirming more silanol nests than those of Rh/S1-OH-5. Rh/S1-OH-20 showed an obvious signal at 3734 cm^{-1}, which might be due to the combination of the enhanced signals at 3726 and 3745 cm^{-1} due to more organosilanes in the synthesis gel. These results confirm that the silanol concentration in the zeolite crystals could be reasonably adjusted by changing the organosilane amount in the starting gels.

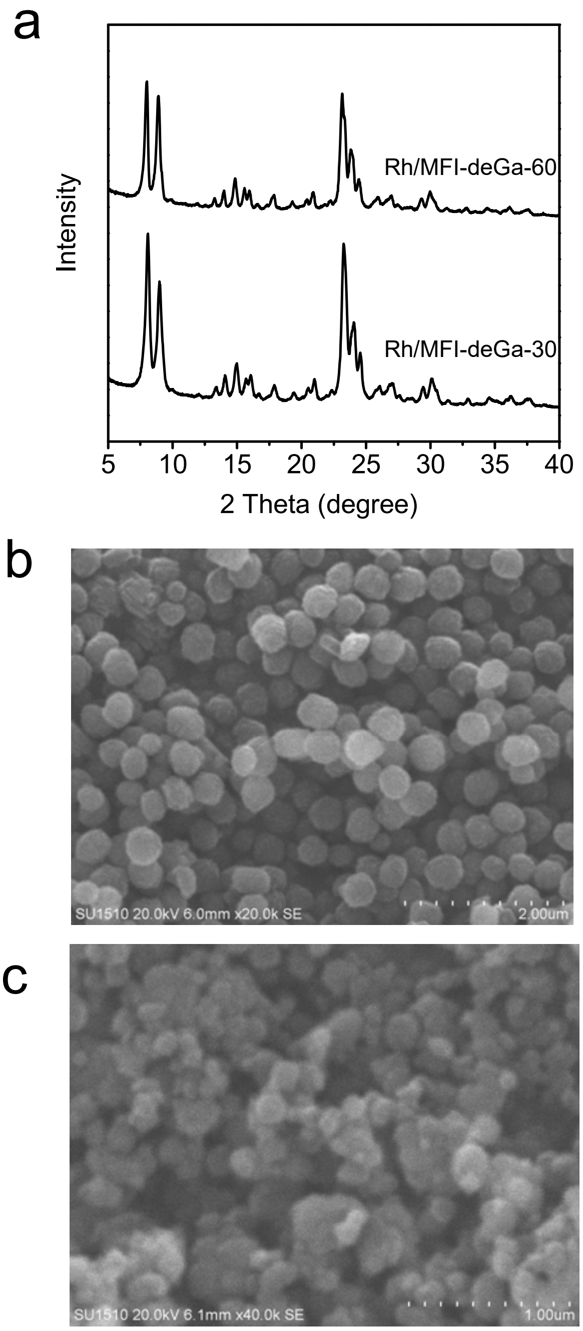

Supplementary Fig. 29. (a) XRD patterns and SEM images of the (b) Rh/MFI-deGa-30 and (c) Rh/MFI-deGa-60 catalysts.

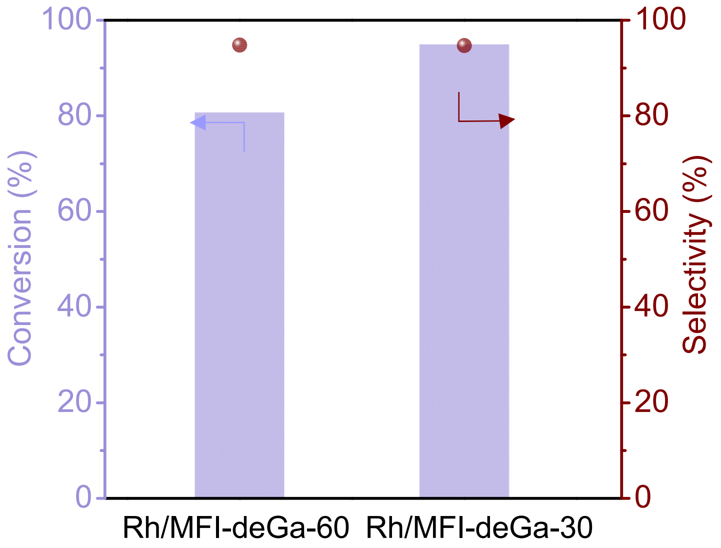

Supplementary Fig. 30. Data characterizing the catalytic performances of the Rh/MFI-deGa-30 and Rh/MFI-deGa-60 catalysts in the hydroformylation of styrene.

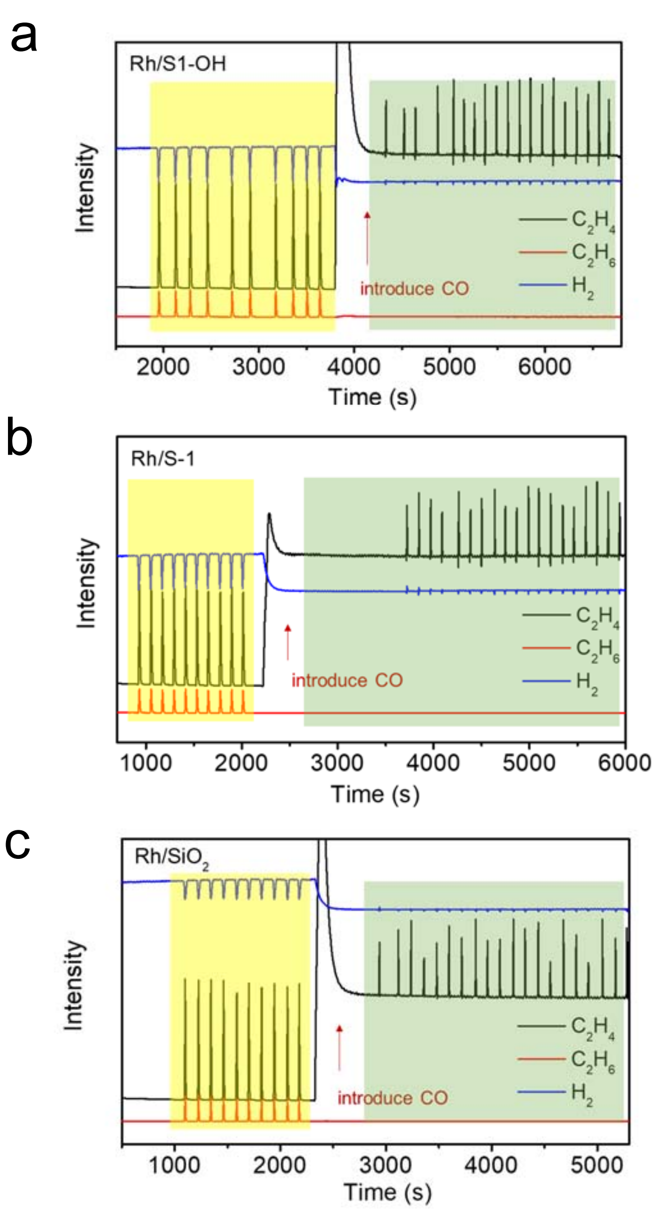

Supplementary Fig. 31. Step-dosing experiments over the (a) Rh/S1-OH, (b) Rh/S-1, and (c) Rh/SiO_2 catalysts in ethylene hydrogenation. The catalysts were pretreated with a 10% H_2/Ar flow at a feed rate of 30.0 ml min^{-1} at 200 °C for 1h. Then, 20 vol% C_2H_4/N_2 with a feed rate at 20 ml min^{-1} was subsequently introduced at 110 °C for the hydrogenation reaction. In this case, 10 vol% CO/N_2 at 20 ml min^{-1} was subsequently introduced into the tube and the MS signals were collected with time.

Note: During the tests, the ethylene was pulsed for multiple times into the catalyst with a continuous hydrogen flow, and the MS detector gave the signals in the effluent after each pulse experiment, thus resulting in multiple peaks. For example, in the test without CO, the ethylene hydrogenation easily occurred, giving the appearance of ethane signal (m/z at 30) and reduce signal of hydrogen (m/z at 2, negative relative to the background line). When CO was introduced with hydrogen, the ethane was undetectable when ethylene was pulsed, accompanied with the extremely weak combustion of hydrogen. These data help to understand the hindered deep hydrogenation with the existence of CO in the feed.

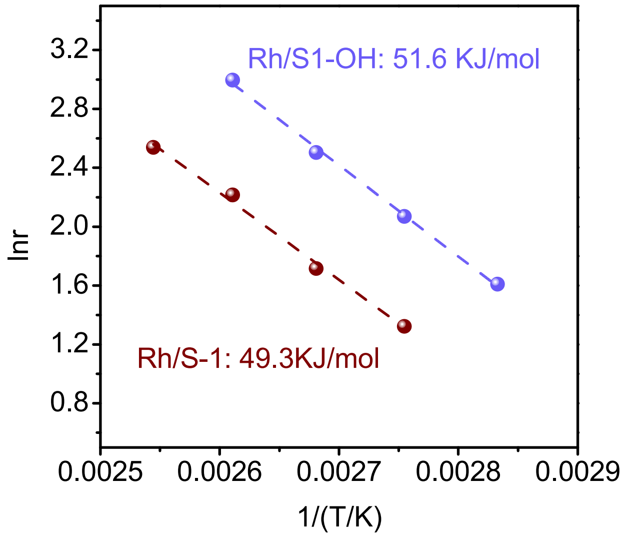

Supplementary Fig. 32. Arrhenius plots of styrene hydroformylation over the Rh/S1-OH and Rh/S-1 catalysts, giving the apparent activation energies for the Rh/S1-OH and Rh/S-1 at 51.6 and 49.3 KJ/mol. Reaction condition: 3 MPa of syngas with molar ratio of CO to H_2 at 1 (molar ratio of CO/H_2/Ar at 45/45/10), 2.5 mmol of styrene were mixed in 5 mL of toluene as solvent, and 30 mg of catalyst, where the styrene conversion was controlled below 20 %.

Note: The apparent activation energies of Rh/S1-OH and Rh/S-1 (51.6 KJ/mol and 49.3 KJ/mol) are similar to those of the general hydroformylation reactions reported previously [14-16].

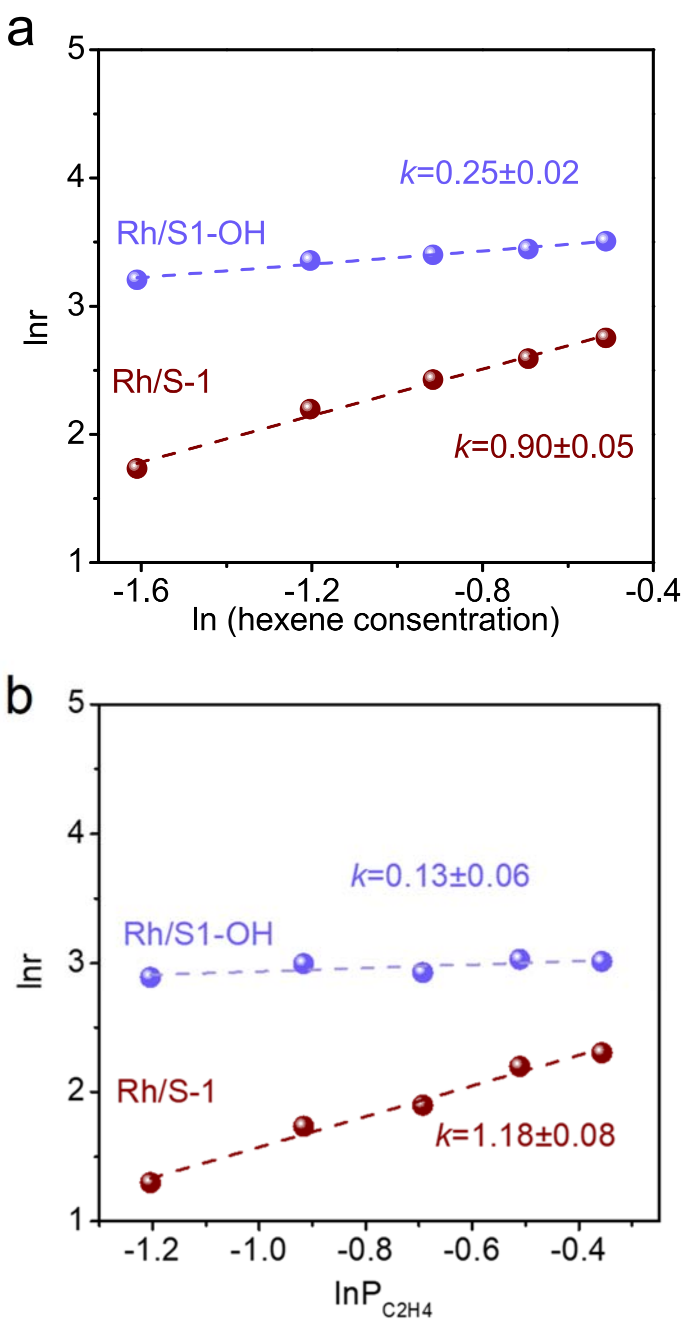

Supplementary Fig. 33. Kinetic reaction orders to (a) hexene and (b) ethylene in Rh/S-1 and Rh/S1-OH catalyzed hydroformylation.

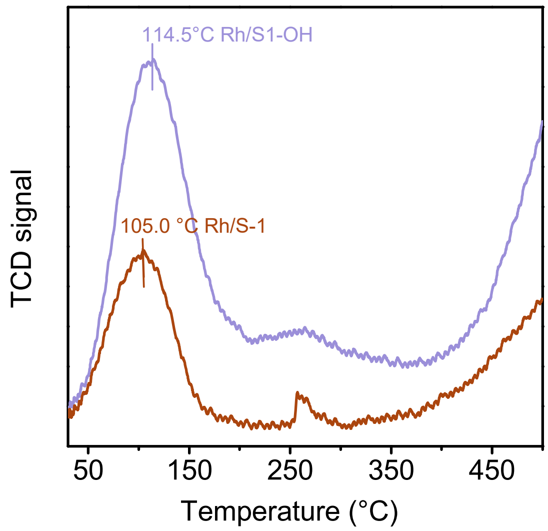

Supplementary Fig. 34. Temperature-programmed desorption tests of ethylene on the Rh/S1-OH and Rh/S-1 samples.

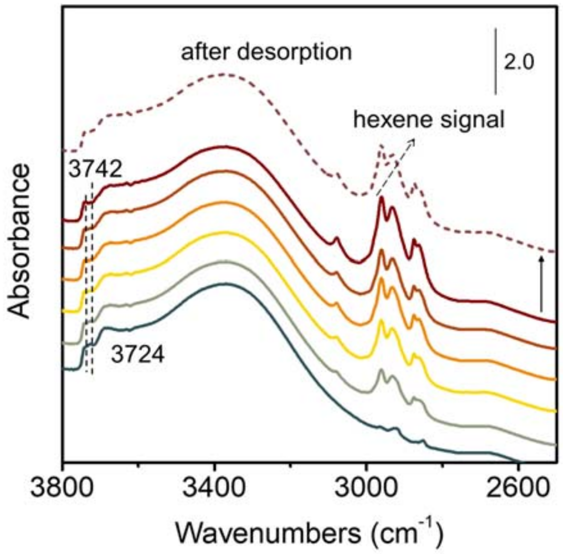

Supplementary Fig. 35. *In situ* hexene-adsorption FTIR spectra over the Rh/S1-OH catalyst.

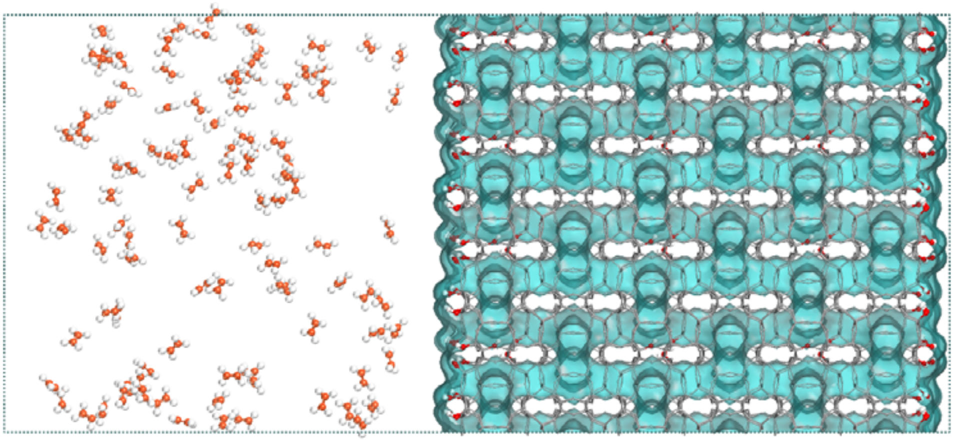

Supplementary Fig. 36. Initial structure of 100 ethylene molecules (C atoms in orange, H atoms in white) adsorbed in gas phase near the S1-OH zeolite surface (grey framework with hydroxyl group in red-white).

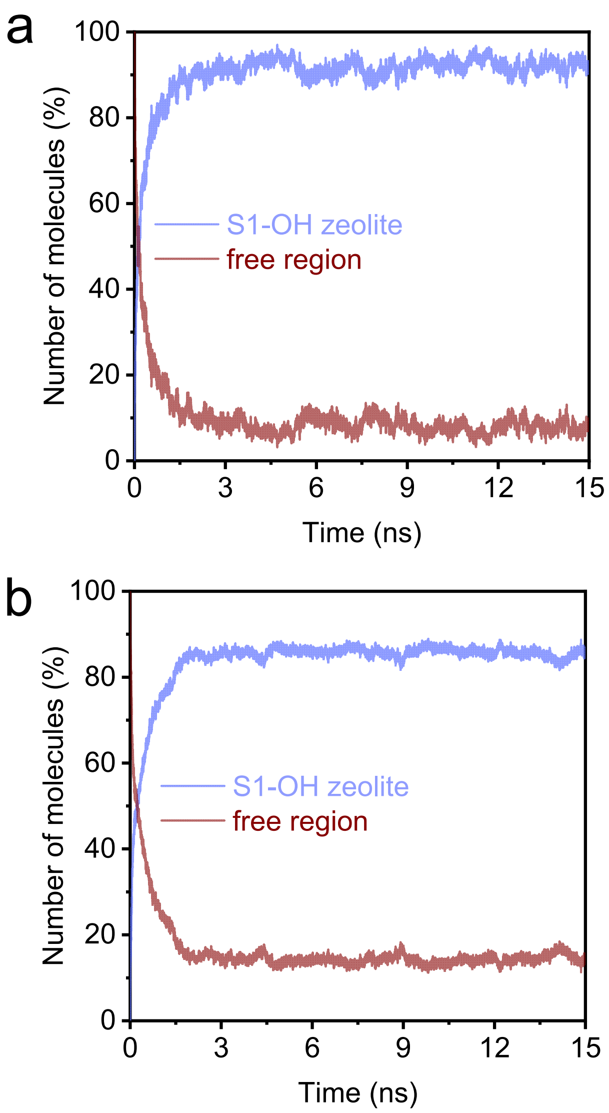

Supplementary Fig. 37. Percentage of ethylene molecules in free region (simulating the homogeneous system) and S1-OH zeolite during the diffusion process with the initial number of ethylene molecules at (a) 50 and (b) 200.

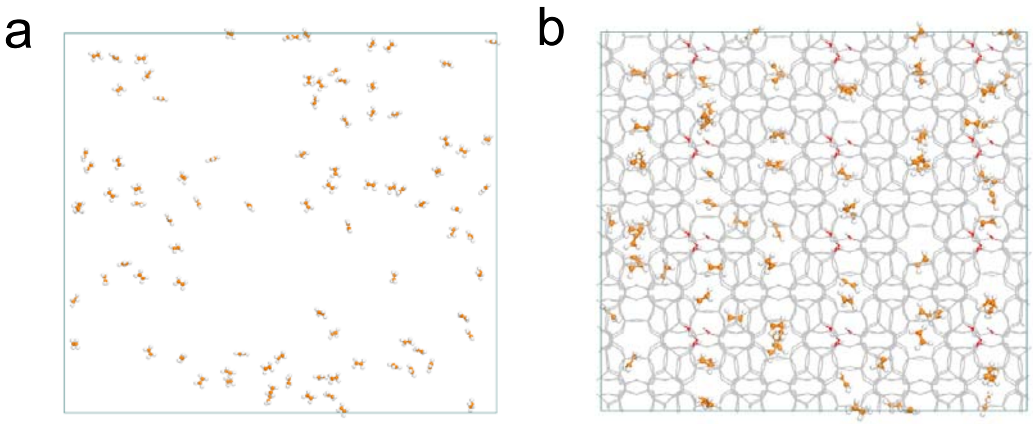

Supplementary Fig. 38. Periodic structure of ethylene in (a) free region (simulating the homogeneous system, 120.5 × 120.0 × 105.1 Å^3) and (b) S1-OH zeolite (60.3 × 59.2 × 52.6 Å^3).

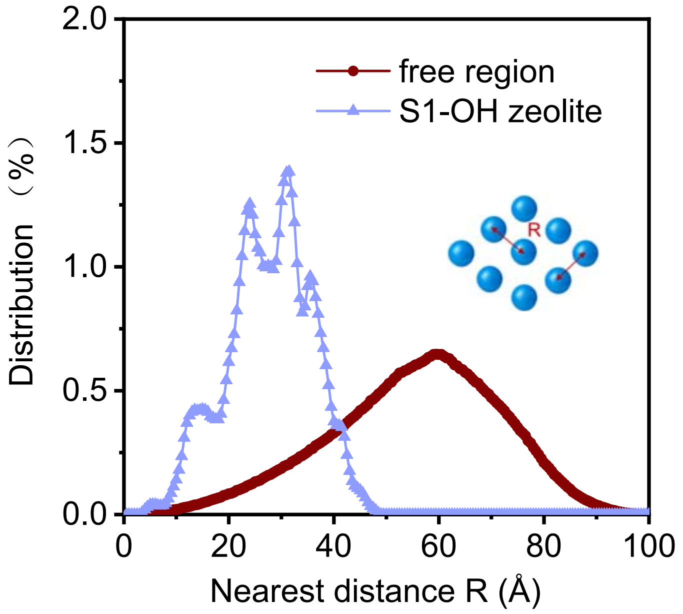

Supplementary Fig. 39. Distribution of two nearest-neighbor ethylene molecules as a function of distance.

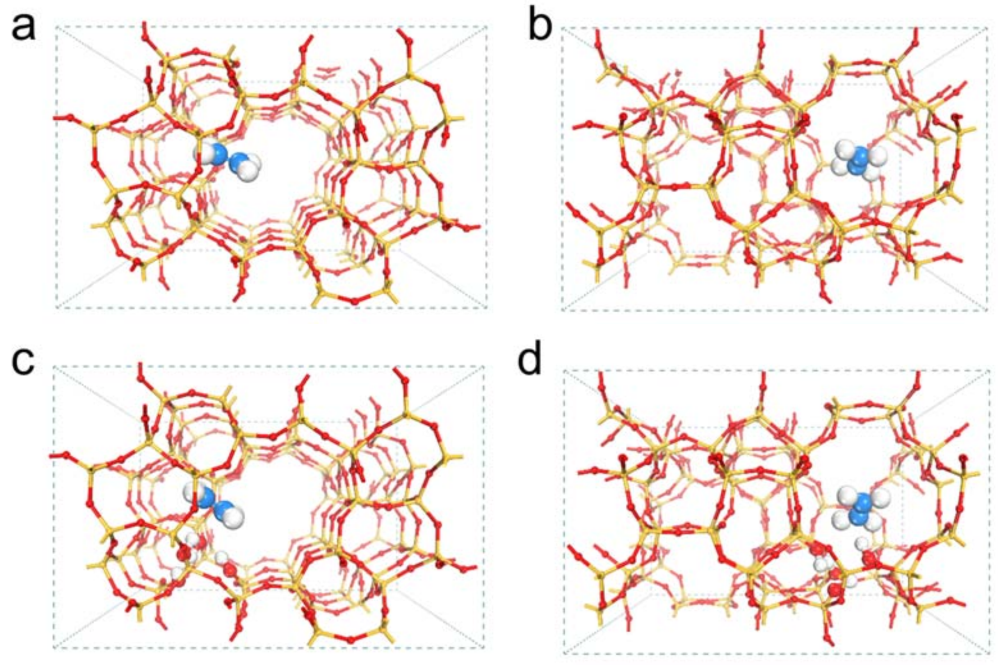

Supplementary Fig. 40. Structures showing ethylene adsorption within the micropores of MFI structures. Siliceous MFI (S1) along (a) Y and (b) X directions, silanol-modified siliceous MFI (S1-OH) along (c) Y and (d) X directions.

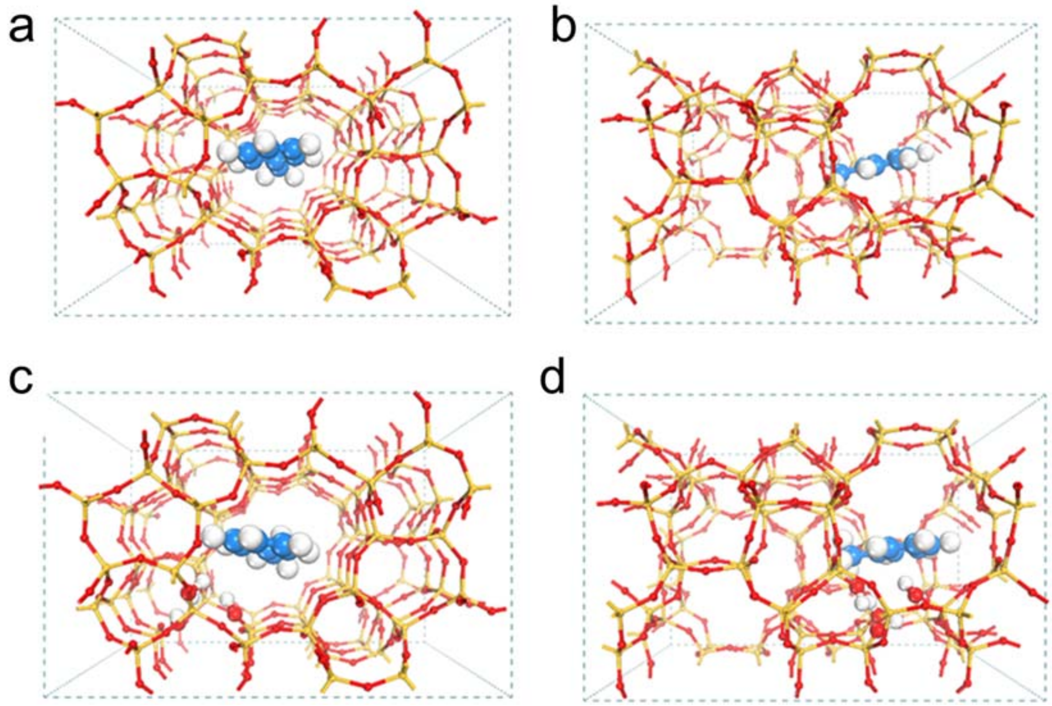

Supplementary Fig. 41. Structures showing toluene adsorption within the micropores of MFI structures. Siliceous MFI (S1) along (a) Y and (b) X directions, silanol-modified siliceous MFI (S1-OH) along (c) Y and (d) X directions.

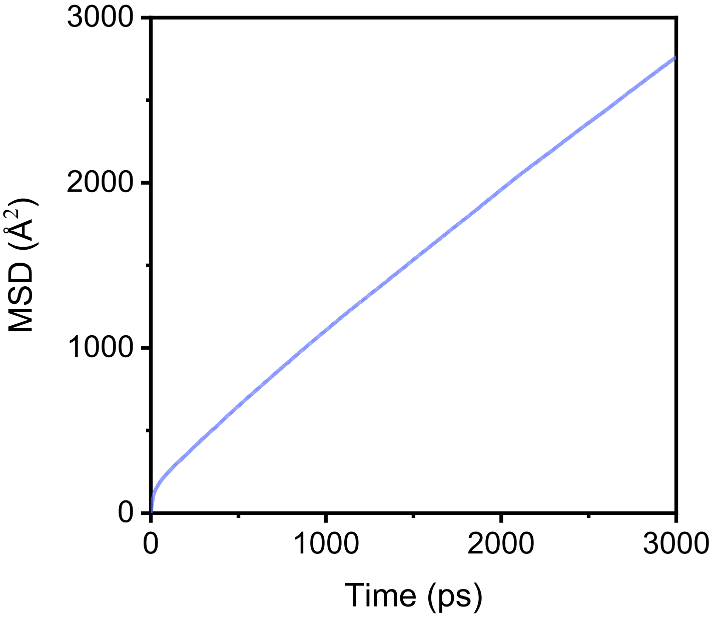

Supplementary Fig. 42. Mean square displacement (MSD) of ethylene molecules diffusing in the S1-OH zeolite.

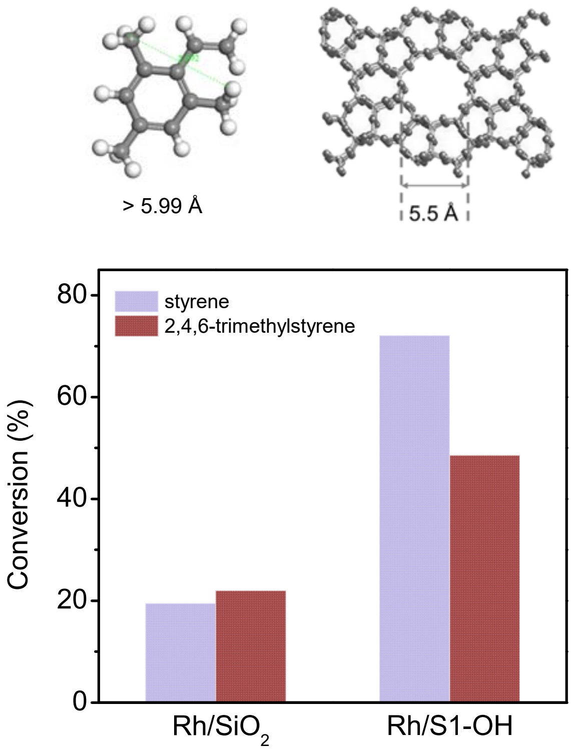

Supplementary Fig. 43. Data characterizing catalytic performances of the Rh/SiO_2 and Rh/S1-OH catalysts in the hydroformylation of styrene and 2,4,6-trimethylstyrene. Reaction conditions: 2.5 mmol of substrate, 5 mL of toluene, 110 °C, 3.0 MPa of syngas with a molar ratio of CO to H_2 at 1 (molar ratio of CO/H_2/Ar at 45/45/10), 30 mg of catalysts, reaction time at 4h for Rh/SiO_2, and 2.5 h for the Rh/S1-OH.

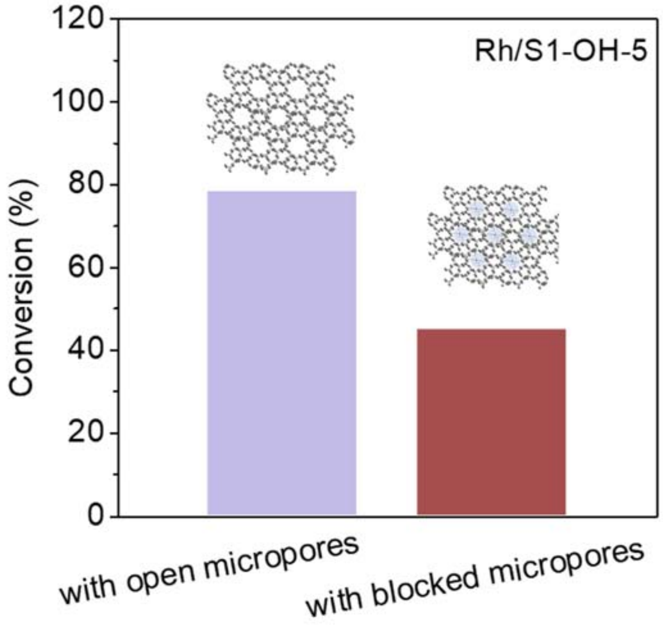

Supplementary Fig. 44. Data showing the catalytic performances of various catalysts in the hydroformylation of styrene. Reaction conditions: 2.5 mmol of styrene, 5 mL of toluene, 110 °C, 3.0 MPa of syngas with a molar ratio of CO to H_2 at 1 (molar ratio of $CO/H_2/Ar$ at 45/45/10), 30 mg of catalysts.

Supplementary Table 1. Data showing the Rh loadings on different catalysts.

Entry	Catalyst	Rh loading (wt%)[a]
1	Rh/S1-OH	0.060
2	Rh/S-1	0.058
3	Rh/ZSM-5	0.055
4	Rh/SiO_2	0.069.
5	Rh/γ-Al_2O_3	0.062
6	Rh/TiO_2	0.071
7	Rh/CeO_2	0.061
8	Rh/S1-OH-used	0.059

[a] By ICP analysis, the error bound was ±0.005%.

Supplementary Table 2. TOF comparison of various catalysts in hydroformylation of olefins.

Entry	Catalyst	TOF (h^{-1})	Reaction conditions	Ref.
1.	Rh/S1-OH	50000	3.0 MPa of syngas with molar ratio of CO to H_2 at 1 (molar ratio of $CO/H_2/Ar$ at 45/45/10), 15mg of catalyst, 20 mmol of styrene 110 °C, 4 h	This work
2	Rh/S-1	4525	3.0 MPa of syngas with molar ratio of CO to H_2 at 1 (molar ratio of $CO/H_2/Ar$ at 45/45/10), 15mg of catalyst, 20 mmol of styrene 110 °C, 4 h	This work
3	$RhCl(PPh_3)_3$	15600	3.0 MPa of syngas with molar ratio of CO to H_2 at 1 (molar ratio of $CO/H_2/Ar$ at 45/45/10), 20 mmol of styrene 110 °C, 4 h	This work
4	$RhCl_3$	4700	3.0 MPa of syngas with molar ratio of CO to H_2 at 1 (molar ratio of $CO/H_2/Ar$ at 45/45/10), 20 mmol of styrene 110 °C, 4 h	This work
5	0.006%Rh_1/ZnO-nw	3333	0.8 MPa CO, 0.8 MPa H_2, 100°C, 20 mg of catalyst, 2.5 mmol of styrene	17
6	Rh-SLMC-0.1%	6367	5.0 mmol of styrene; styrene/Rh = 51450 4.0 ml of toluene; 2.0 MPa H_2, 2.0 MPa CO,100°C, 8 h	18
7	Rh/S-g-C_3N_4	9000	20 mg of catalyst, 1.5 mL of styrene, 3 h, 100 °C, 6.0 MPa of syngas (CO/H_2 = 1).	19
8	Rh/4%B-g-C_3N_4	12000	0.01 g of catalyst, toluene: 20 mL, styrene: 1.0 mL, reaction time: 3 h, 100 °C, syngas (CO/H_2 = 1): 6.0 MPa	20
9	$HRh(CO)(PPh_3)_2$	2400-36000	80-130 °C<br>0.8-2 MPa syngas	21
10	$HRh(CO)_4$	9000	100-150 °C<br>10-30 MPa syngas	21
11	Rh/PSAs	1158	5.0 mmol 1-olefin, 10 mL of H_2O, 100 °C, CO/H_2 = 1/1 (2.0 MPa), S/C = 3000, 4 h.	22
12	RhZn/SBA-15	3090	0.5 mmol of olefin, 2.0 mL of toluene, 30 bar of CO/H_2 (1/1), 100 °C, 2 h	23
13	Rh@POP-PTBA-HA-50	801	10 mmol of 1-octene, 25 mg of catalyst S/C = 11440, CO/H_2 = 3/3 MPa, CH_3CN = 4 mL, 120 °C, 12 h	24

14	Rh/CPOL-BP&P	11200	10 mg of catalyst, 24 h, 2 MPa (CO/H_2 = 1/1), GHSV = 8000 h^{-1}, 80 °C	25
15	Rh/CoO	2065	H_2, CO, propene (H_2/CO/propene = 1/1/1) 100 °C, 2 h,	26
16	0.6Rh0.23Co/SiO_2	2790	180 °C, 1 MPa, GHSV = 6000 mL/(g_{cat}·h), $H_2/CO/C_2H_4/N_2$ = 1/1/1/1.	27
17	Kx^Rh@S-1	7328	catalyst 0.1 g, propylene 0.5 MPa, 1-hexene 1 g, CO: H2 = 1:1, 5 MPa, toluene 5 mL, 85 °C, 3 h.	28
18	Rh@Y	6567	120 °C, 1-hexene, 6 MPa syngas	29
19	0.18% Rh/B-TNTs	18458	0.40 g catalysts, 65 mL toluene, 5 mL styrene, gas (CO/H_2): 6.0 MPa, 80 °C.	30
20	0.5% Rh/Al_2O_3	3189	4.8 mmol of Styrene, S/Rh = styrene/Rh molar ratio from 500 to 100000; 8MPa of syngas, 10 ml of toluene, 80 °C.	31
21	[Rh(acac)$(CO)_2$]/ Biphephos	8200	120 °C, 3 MPa of syngas, 1-hexene	32
22	[Co(acac)(DPPBz)] (BF_4)	6192	160 °C 1 mM catalyst (61 ppm Co), 1 M 1-hexene, 0.1 M heptane standard, dimethoxytetraglyme solvent, 9 MPa of syngas	21
23	Rh-silc/tppti	3600	100 °C Rh/P ratio = 1/10, silc runs were evaluated in a 70 mL autoclave at 1500 psi, and biphasic and homogeneous catalyst systems were evaluated in a 300 mL autoclave at 600 psi. 5h	33
24	$Rh(PPh_3)_3$	5000	H_2, CO, propene (H_2/CO/propene = 1/1/1) 100 °C, 2 h.	26
25	Rh/(S,S)-Diazaphospholane	19400	80 °C, vinyl acetate/Rh molar ratio = 100000, (S,S)-1/Rh=1.2, 4 mL of vinyl acetate, 5 h	34

Supplementary Table 3. Data characterizing the performances of the Rh/S1-OH catalyst in the hydroformylation of various substrates.

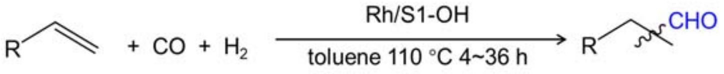

Entry [a]	Substrate	Product	Conversion (%)	Selectivity (%)	l/b
**1**	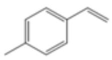	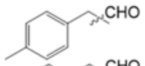	99.0	98.2	1.10
**2**[b]	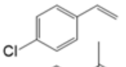	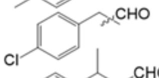	95.0	99.0	1.53
**3**[c]	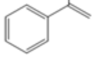	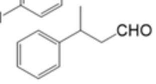	83.4	96.5	---
**4**	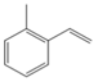	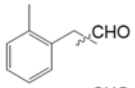	92.0	97.1	2.93
**5**	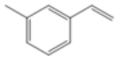	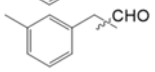	97.0	93.4	1.25
**6**	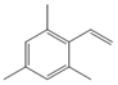	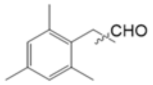	81.0	94.7	10.4
**7**	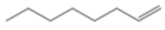	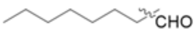	82.3	97.8	0.65

[a] Reaction conditions: syngas with a molar ratio of CO to H_2 at 1 (molar ratio of CO/H_2/Ar at 45/45/10), 30 mg of catalyst, 2.5 mmol of substrate, 5 mL of toluene as solvent, butanol as internal standard, 110 °C, 4 h.

[b] Reaction for 12 h.

[c] Reaction for 36 h.

Supplementary Table 4. Parameter of the force fields for zeolites and ethylene in theoretical simulation [11].

Atom type	ε (kcal/mol)	σ (Å)	q (e)
Oh [Si-Oh-H][10]	0.122	3.090	-0.675
Os [Si-Os-Si][9]	0.105	3.300	-0.750
Hs [Si-O-Hs][10]	0.015	0.970	0.400
Sh [Si-O-H][10]	0.093	3.700	1.400[a]
Si [O-Si-O][9]	0.044	2.300	1.500
CH_2 [CH_2-CH_2][8]	0.169	3.675	0.000

[a] The charge has been adjusted to keep the total change equal 0.

Supplementary Table 5. Adsorption energy for various molecule in S1 and S1-OH zeolites.[a]

Molecule	S1 (eV)	S1-OH (eV)
ethylene	-0.38	-0.52
1-hexene	-0.64	-0.78
toluene	-0.91	-0.96

[a] The calculations were performed using the DFT-D3 method at the gamma point with a cutoff energy of 400 eV.

Supplementary References.

1. Ryoo, R., Kim, J., Jo, C., Han, S., Kim, J.-C., Park, H., Han, J., Shin, H. & Shi J. Rare-earth–platinum alloy nanoparticles in mesoporous zeolite for catalysis. *Nature* **585**, 221–224 (2020).
2. Hohwy, M., Jakobsen, H. J., Edén, M., Levitt, M. H. & Nielsen N. C. Broadband dipolar recoupling in the nuclear magnetic resonance of rotating solids: A compensated C7 pulse sequence. *J. Chem. Phys.* **108**, 2686–2694 (1998).
3. Kresse, G. & Furthmuller, J. Efficiency of ab-initio total energy calculations for metals and semiconductors using a plane-wave basis set. *Comput. Mater. Sci.* **6**, 15–50 (1996).
4. Kresse, G. & Furthmuller, G. J. Efficient iterative schemes for ab initio total energy calculations using a plane-wave basis set. *Phys. Rev. B* **54**, 11169–11186 (1996).
5. Blochl, P. E. Projector augmented-wave method. *Phys. Rev. B* **50**, 17953–17979 (1994).
6. Kresse, G. & Joubert, D. From ultrasoft pseudopotentials to the projector augmented-wave method. *Phys. Rev. B* **59**, 1758–1775 (1999).
7. Perdew, J. P., Burke, K. & Ernzerhof, M. Generalized gradient approximation made simple. *Phys. Rev. Lett.* **77**, 3865–3868 (1996).
8. Wick, C. D., Martin, M. G. & Siepmann, J. I. Transferable potentials for phase equilibria. 4. united-atom description of linear and branched alkenes and alkylbenzenes. *J. Phys. Chem. B* **104**, 8008–8016 (2000).
9. Bai, P., Tsapatsis, M. & Siepmann, J. I. TraPPE-zeo: Transferable potentials for phase equilibria force field for all-silica zeolites. *J. Phys. Chem. C* **117**, 24375–24387 (2013).
10. Emami, F. S., Puddu, V., Berry, R. J., Varshney, V., Patwardhan, S. V., Perry, C. C. & Heinz, H. Force field and a surface model database for silica to simulate interfacial properties in atomic resolution, *Chem. Mater.* **26**, 2647–2658 (2014).
11. Wang, C. T., Fang, W., Liu Z. Q., Wang, L., Liao, Z. W., Yang, Y. R. Li, H. J., Liu, L., Zhou, H. Qin, X. D, Xu, S. D., Chu, X. F., Wang, Q. Y., Zheng, A. M., & Xiao, F.-S. Fischer–Tropsch synthesis to olefins boosted by MFI zeolite nanosheets. *Nat. Nanotechnol.* **17**, 714-720 (2022).
12. Smith, W. & Forester, T. R. DL_POLY_2.0: A general-purpose parallel molecular dynamics simulation package. *J. Mol. Graph.* **14**, 136–141 (1996).
13. Smit, B. & Maesen, T. L. M. Molecular simulations of zeolites: Adsorption, diffusion, and shape selectivity. *Chem. Rev.* **108**, 4125−4184 (2008).
14. Ro, I., Qi, J., Lee, S., Xu, M., Yan, X., Xie, Z., Zakem, G., Morales, A.,Chen, J. G., Pan, X., Vlachos, D. G., Caratzoulas, S. & Christopher, P. Bifunctional hydroformylation on heterogeneous Rh-WOx pair site catalysts. *Nature* **609**, 287–292 (2022).
15. Shylesh, S., Hanna, D., Mlinar, A., Kong, X., Reimer, J. A. & Bell, A. T. *In situ* formation of Wilkinson-type hydroformylation catalysts: insights into the structure, stability, and kinetics of triphenylphosphine- and xantphos-modified Rh/SiO_2. *ACS Catal.* **3**, 348−357 (2013).
16. Ro, I., Xu, M., Graham, G. W., Pan, X. & Christopher, P. Synthesis of heteroatom Rh−ReOx atomically dispersed species on Al_2O_3 and their tunable catalytic reactivity in ethylene hydroformylation. *ACS Catal.* **9**, 10899−10912 (2019).
17. Lang, R., Li, T., Matsumura, D., Miao, S., Ren, Y., Cui, Y., Tan, Y., Qiao, B., Li, L., Wang, A., Wang, X. & Zhang, T. Hydroformylation of olefins by a rhodium single-atom catalyst with activity comparable to $RhCl(PPh_3)_3$. *Angew. Chem. Int. Ed.* **55**, 16054 –16058 (2016).
18. Sun, H., Guo, W., Liu, J., Feng, Z., Li, R., Zhou, X. & Huang, J. Supported rhodium liquid metal catalysts for the hydroformylation of olefins. *Appl. Organometal Chem.* **32**, e4555 (2018).
19. Shi, Y., Lu, Y., Ren, T., Li, J., Hu, Q., Hu, X., Zhu, B. & Huang, W. Rh particles supported on sulfated g-C3N4: a highly efficient and recyclable heterogeneous catalyst for alkene hydroformylation. *Catalysts* **10**, 1359 (2020).

20. Shi, Y., Ji, G., Hu, Q., Lu, Y., Hu, X., Zhu, B. & Huang, W. Highly uniform Rh nanoparticles supported on boron doped g-C_3N_4 as a highly efficient and recyclable catalyst for heterogeneous hydroformylation of alkenes. *New J. Chem.* **44**, 20−23 (2020).
21. Hood, D. M., Johnson, Ryan A., Carpenter. A. E., Younker, J. M., Vinyard. D. J. & Stanley. G. G. Highly active cationic cobalt(II) hydroformylation catalysts. *Science* **367**, 542–548 (2020).
22. Dong, K., Sun, Q., Tang, Y., Shan, C., Aguila, B., Wang, S., Meng, X., Ma, S. & Xiao, F.-S. Bio-inspired creation of heterogeneous reaction vessels via polymerization of supramolecular ion pair. *Nat. Commun.* **10**, 3059 (2019).
23. Chen, M., Gupta, G., Ordonez, C. W., Lamkins, A. R., Ward, C. J., Abolafia, C. A., Zhang, B., Roling, L. T. & Huang, W. Intermetallic nanocatalyst for highly active heterogeneous hydroformylation. *J. Am. Chem. Soc.* **143**, 20907−20915 (2021).
24. Zhao, K., Wang, H., Wang, X., Li, T., Dai, X., Zhang, L., Cui, X. & Shi, F. Confinement of atomically dispersed Rh catalysts within porous monophosphine polymers for regioselective hydroformylation of alkenes. *J. Catal.* **401,** 321-330 (2021).
25. Wang, Y., Yan, L., Li, C., Jiang, M., Wang, W. & Ding, Y. Highly efficient porous organic copolymer supported Rh catalysts for heterogeneous hydroformylation of butenes. *Appl.Catal. A-Gen.* **551,** 98–105 (2018).
26. Wang, L., Zhang, W., Wang, S., Gao, Z., Luo Z., Wang, X., Zeng, R., Li, A., Li, H., Wang, M., Zheng, X., Zhu, J., Zhang, W., Ma, C., Si, R. & Zeng, J. Atomic-level insights in optimizing reaction paths for hydroformylation reaction over Rh/CoO single-atom catalyst. *Nat. Commun.* **7**, 14036 (2016).
27. Huang, N., Liu, B., Lan. X. & Wang, T. Insights into the bimetallic effects of a RhCo catalyst for ethene hydroformylation: experimental and DFT investigations. *Ind. Eng. Chem. Res.* **59**, 18771−18780 (2020).
28. Zhang, J., Sun, P., Gao, G., Wang, J., Zhao, Z., Muhammad, Y. & Li, F. Enhancing regioselectivity via tuning the microenvironment in heterogeneous hydroformylation of olefins. *J. Catal.* **387,** 196–206 (2020).
29. Shang, W., Qin, B., Gao, M., Qin X., Chai, Y., Wu, G., Guan, N., Ma, D., & Li, L. Efficient heterogeneous hydroformylation over zeolite-encaged isolated rhodium ions. *CCS Chem.* **0** 1−14 (2022).
30. Shi, Y., Hu, X., Chen, L., Lu, Y.,Zhu, B., Zhang, S. & Huang, W. Boron modified TiO_2 nanotubes supported Rh-nanoparticle catalysts for highly efficient hydroformylation of styrene. *New J. Chem.* **41**, 6120–6126 (2017).
31. Alini, S., Bottino, A., Capannelli, G., Comite, A. & Paganelli, S. Preparation and characterisation of Rh/Al_2O_3 catalysts and their application in the adiponitrile partial hydrogenation and styrene hydroformylation. *Appl. Catal. A Gen.* **292**, 105–112 (2005).
32. Vogl, C., Paetzold, E., Fischer, C. & Kragl, U. Highly selective hydroformylation of internal and terminal olefins to terminal aldehydes using a Rhodium-BIPHEPHOS-catalyst system. *J. Mol. Catal. A Chem.* **232**, 41–44 (2005).
33. Mehnert, C. P., Cook, R. A., Dispenziere, N. C. & Afeworki, M. Supported ionic liquid catalysis - a new concept for homogeneous hydroformylation Catalysis. *J. Am. Chem. Soc.* **124**, 12932–12933 (2002).
34. Thomas, P. J., Axtell, A. T., Klosin, J., Wei, P., Rand, C. L.; Clark, T. P.; Landis, C. R. & Abboud, K. A. Asymmetric hydroformylation of vinyl acetate: application in the synthesis of optically active isoxazolines and imidazoles. *Org. Lett.* **9**, 2665–2668 (2007).
